# Supplementary material for: Image-based modeling of kidney branching morphogenesis reveals GDNF-RET based Turing-type mechanism and pattern-modulating WNT11 feedback
Source: Nat Commun. 2019 Jan 16;10:239. doi: 10.1038/s41467-018-08212-8 (PMC6484223; doi:10.1038/s41467-018-08212-8)
Supplement: Supplementary file 1 — Supplementary Information [file 41467_2018_8212_MOESM1_ESM.pdf]

SUPPLEMENTARY MATERIAL

Image-Based Modeling of Kidney Branching Morphogenesis reveals core GDNF-RET based Turing-type Mechanism and pattern-modulating WNT11 Feedback

Denis Menshykau[1,2*], Odyssé Michos[1,2,3], Christine Lang[1,2], Lisa Conrad[1,2], Andrew P. McMahon[3], Dagmar Iber[1,2,*]

1 Department for Biosystems Science and Engineering, ETH Zurich, Mattenstrasse 26, 4058 Basel, Switzerland

2 Swiss Institute of Bioinformatics, Mattenstrasse 26, 4058 Basel, Switzerland

3 Department of Stem Cell Biology and Regenerative Medicine, Eli and Edythe Broad Center for Regenerative Medicine and Stem Cell Research, University of Southern California Keck School of Medicine, Los Angeles, CA 90089, USA

∗ To Whom Correspondence should be addressed. E-mail: dagmar.iber@bsse.ethz.ch (D.I.); denis.menshykau@gmail.com (D.M.)

Supplementary Notes

1 The Necessary Conditions for the Turing Mechanism

For the convenience for the reader, we summarise well established details about the Turing mechanism. The text follows [1, 2].

1.1 The Turing Mechanism

In this section we summarize the criteria for the emergence of a Turing pattern in reaction-diffusion systems with two species. We consider a general case of the ligand-receptor dynamics (Eq 1) systems in the form

$$\begin{aligned} \frac{\partial R}{\partial \tau} &= F(R, L) + D_R \Delta R \\ \frac{\partial L}{\partial \tau} &= G(R, L) + D_L \Delta L \end{aligned} \qquad \text{(Supplementary Equation-1)}$$

defined on $(0, \infty) \times \Omega$ (with a given spatial domain $\Omega \subset \mathbb{R}^n$) subject to boundary and initial conditions, where the space and time-dependent variables R and L represent concentrations and the reaction kinetic terms F and G are generally nonlinear functions. After suitable changes of variables and nondimensionalization Eq. (Supplementary Equation-1) can be transformed into the dimensionless system

$$\begin{aligned} R_t &= \gamma f(R, L) + \Delta R \\ L_t &= \gamma g(R, L) + d \Delta L, \end{aligned} \qquad \text{(Supplementary Equation-2)}$$

where $(\cdot)_t$ represents the time derivative, t is the rescaled time variable, d denotes (or is proportional to) the quotient of the diffusion coefficients D_R and D_L and $\gamma = const \cdot l^2$, where l is a typical length scale of the domain. To ensure the uniqueness of the solution we endow system (Supplementary Equation-2) with initial and boundary conditions. We will use homogeneous Neumann boundary condition of the

form

$$(\mathbf{n} \cdot \nabla) \begin{pmatrix} R \\ L \end{pmatrix} = 0 \quad \text{on } [0, \infty) \times \partial\Omega$$

$$R(0, \mathbf{x}) = R_0(\mathbf{x}),\ L(0, \mathbf{x}) = L_0(\mathbf{x}),$$

because they are easy to handle and have a biological interpretation (impermeable boundary). We note, however, that other boundary conditions would not greatly alter the following analysis. A Turing instability appears when a reaction-diffusion system has a stable steady state in the absence of diffusion, which loses its stability in the presence of diffusion such that spatial patterns emerge.

1.2 Linear stability in the absence of diffusion

Let R_0 and L_0 denote the steady state of the diffusion-free system of ordinary differential equations (ODEs)

$$R_t = \gamma f(R, L), \qquad L_t = \gamma g(R, L), \qquad \text{(Supplementary Equation-3)}$$

and linearize the system about (R_0, L_0) by introducing the translated function $\mathbf{w} = (w_1, w_2)^T$ with $w_1 = R - R_0,\ w_2 = R - R_0$. Then the linearized system becomes

$$\mathbf{w}_t = \gamma J \mathbf{w},$$

where

$$J = \begin{pmatrix} f_R & f_L \\ g_R & g_L \end{pmatrix} \Bigg|_{(R_0, L_0)} = \begin{pmatrix} f_R(R_0, L_0) & f_L(R_0, L_0) \\ g_R(R_0, L_0) & g_v(R_0, L_0) \end{pmatrix}$$

is the Jacobian evaluated at the point (R_0, L_0). From now on, we write the partial derivatives evaluated at the steady state without their arguments for brevity. The steady state of the linearized system is stable, i.e. the steady state of system (Supplementary Equation-3) is linearly stable if $\Re\lambda(J) < 0$ for all eigenvalues of J, which for a 2-component system is ensured by the conditions

$$\text{tr}J = f_R + g_L < 0, \qquad \det(J) = f_R g_L - f_R g_L > 0. \qquad \text{(Supplementary Equation-4)}$$

1.3 Diffusion-driven instability

Now let us add diffusion to our system of ODEs and consider the reaction-diffusion system linearized about the steady state $\mathbf{w} = (0,0)^T$, which has the form

$$\mathbf{w}_t = \gamma J \mathbf{w} + D\Delta \mathbf{w}, \qquad \text{(Supplementary Equation-5)}$$

where $D = \text{diag}(1, d)$ is a diagonal matrix containing the diffusion coefficients of the nondimensionalized system (Supplementary Equation-2). We look for a solution of the form

$$\mathbf{w}(t, \mathbf{x}) = \sum_k \mathbf{C}_k e^{\lambda_k t} \mathbf{W}_k(\mathbf{x}), \qquad \text{(Supplementary Equation-6)}$$

where the exponents λ_k determine the temporal growth of the solution and the time-independent functions $\mathbf{W}_k$ are the solutions of the elliptic eigenvalue problem

$$\Delta \mathbf{W}_k + k^2 \mathbf{W}_k = 0, \qquad (\mathbf{n} \cdot \nabla)\mathbf{W}_k = 0. \qquad \text{(Supplementary Equation-7)}$$

For instance, in one dimension on the interval $[0, L]$ the eigenvalues are $k = n\pi/L$ ($n = 0, 1, 2, \ldots$), also called wavenumbers, and the eigenfunctions are $W(x) = \cos(n\pi x/L) = \cos(kx)$. The constants $\mathbf{C}_k = (C_k^{(1)}, C_k^{(2)})^T$ are the Fourier-coefficients of the initial conditions.

Inserting equation (Supplementary Equation-6) into equation (Supplementary Equation-7) and using the fact that the set of eigenfunctions of the Laplace operator $\{\mathbf{W}_k\}$ forms a complete orthonormal system, we obtain as linearized system

$$\mathbf{w}_t = \gamma J \mathbf{w} + D k^2 \mathbf{w} \qquad \text{(Supplementary Equation-8)}$$

for each wavenumber k. Writing

$$\det(\lambda I - \gamma J + k^2 D) = 0,$$

where $I = I_2$ is the 2-by-2 identity matrix, we obtain the eigenvalues $\lambda = \lambda_k$ of the matrix $M = \gamma J - k^2 D$. Expanding the above determinant, we obtain that λ_k is the root of the second order polynomial equation

$$\lambda^2 + \lambda\big(k^2(1+d) - \gamma(f_R + g_L)\big) + dk^4 - \gamma(df_R + g_L)k^2 + \gamma^2(f_R g_L - f_L g_R) = 0.$$

Since we look for unstable solutions, we require that $\Re(\lambda_k) > 0$ for some $k \neq 0$. This means that either the coefficient of λ and/or the constant term must be negative. Since the steady state is required to be linearly stable in the absence of diffusion (which corresponds to the case $k = 0$), we must have $k^2(1+d) - \gamma(f_R + g_L) > 0$. Hence, to obtain a λ with positive real part in the presence of diffusion we require

$$h(k^2) := dk^4 - \gamma(df_R + g_L)k^2 + \gamma^2(f_R g_L - f_L g_R) < 0$$

for some nonzero wavenumber k. Since we require $f_R g_L - f_L g_R > 0$ for linear stability in the absence of diffusion ($k = 0$) (Supplementary Equation-4), it follows that $df_R + g_L > 0$ must hold. This condition is not sufficient to ensure the negativity of the function h; an elementary calculation shows that the minimum of h is attained at the point

$$k_{\mathrm{m}}^2 = \gamma \frac{df_R + g_L}{2d},$$

and the minimum value of h is

$$h_{\min} = h(k_{\mathrm{m}}^2) = \gamma^2 \left[(f_R g_L - f_L g_R) - \frac{(df_R + g_L)^2}{4d} \right],$$

which is negative if the expression in the bracket is negative.

In summary, the well-known conditions (see [2, Sec. 2.3]) for which a reaction-diffusion system with two species exhibits a Turing instability are as follows:

$$\begin{gathered} f_R + g_L < 0, \quad f_R g_L - f_L g_R > 0, \\ df_R + g_L > 0, \quad (df_R + g_L)^2 - 4d(f_R + g_L - f_L g_R) > 0, \end{gathered} \qquad \text{(Supplementary Equation-9)}$$

where all partial derivatives are evaluated at the steady state (R_0, L_0). We note that it is possible that these conditions are satisfied, but that no pattern emerges. This is the case when h is not negative for any k within the discrete set of wavenumbers, and only takes a negative value in between two of these discrete wavenumbers. The distance between wavenumbers shrinks as γ is increased, and in the limit of infinite γ the spectrum of k is continuous. Since γ is related to the size of the spatial domain, it follows that on

small domains pattern formation may not happen, while on a sufficiently increased domain patterns may be observed.

1.4 The Necessary Conditions for a Turing Instability

The necessary conditions for a Turing instability follow from (Supplementary Equation-9). Thus,

i) A difference in the diffusion coefficients, $d > 1$.

   Substituting $d = 1$ into the equation (Supplementary Equation-9) we obtain $f_R + g_L > 0$ and $f_R + g_L < 0$, which cannot be both true. Therefore a difference in the diffusion coefficients is a necessary condition for a Turing instability.

ii) The presence of the positive feedback of the ligand-receptor signalling on the receptor abundance, i.e. $v > m\mu$ in Eq 1. In the absence of a sufficiently strong positive feedback Eq 1 can be written as:

$$\begin{aligned} R_t &= \Delta R + \gamma(a - R - R^2 L) \\ L_t &= D\Delta L + \gamma(b - R^2 L) \end{aligned} \qquad \text{(Supplementary Equation-10)}$$

   In this case, the condition $df_R + g_L > 0$ cannot be fulfilled as both $f_R = -R_0 - 2R_0L_0 \leq 0$ and $g_L = -R_0^2 \leq 0$.

iii) Cooperative interactions between the receptor and the ligand ($m \neq n$ when $m + n = 2$). If the ligand-receptor complex stoichiometry is one to one, then the ligand-receptor interactions are given by the following set of equations:

$$\begin{aligned} R_t &= \Delta R + \gamma(a - R + RL) \\ L_t &= D\Delta L + \gamma(b - RL) \end{aligned} \qquad \text{(Supplementary Equation-11)}$$

   Also, in this case not all the necessary conditions for the Turing instability can be fulfilled simultaneously.

2 Convergence and Accuracy of the Computational Method

As described in the Methods section we sampled the parameter space for four alternative models (T1-T4) from the log-normal distribution (Supplementary Figures 3 (wt), 10 (FF) and 11 (FGS)).

2.1 Complete Ligand-receptor based Model with Architecture for Turing Mechanism, T1.

Supplementary Figures 3C (wt), 10C (FF) and 11C (FGS) show that for the ligand-receptor based model, T1, the minimum value of the global deviation, Δ_{g} is observed within the sampled parameter interval and therefore we conclude that the sampled parameter range is sufficiently large. To confirm that the calculated value of the deviation, Δ, is accurate and is not affected by numerical errors we conducted a convergence test (Supplementary Figures 19A (wt), 10C (FF) and 11C (FGS)). We observed that for the ligand-receptor model, T1, the simulation converges as the mesh size decreases (Supplementary Figures 19A (wt), 20A (FF) and 21A (FGS)). The typical value of the mesh elements in the simulations is 1, and the estimated relative accuracy of the deviation Δ is 0.5% or higher.

2.2 Ligand-receptor based Model without Positive Feedback, T2.

Supplementary Figure 3D (wt), 10D (FF) and 11D (FGS) show that for the ligand-receptor based model without positive feedback, T2, the minimum value of deviation, Δ, decreases as the value of parameter a increases. Large values of the parameter a lead to steep concentration gradients at the epithelium-mesenchyme border, which makes the calculation inaccurate. To estimate the accuracy of the computation, we ran a convergence test (Supplementary Figures 19B (wt), 20B (FF), 21B (FGS)). The convergence test shows that the deviation, Δ, reaches the limiting value as the parameter a increases. However, a fine mesh size of 0.1 is necessary to accurately calculate the limiting value of the deviation, Δ on the domain with a typical length scale of 100. The minimum value of the global deviation, Δ_{g}, which was calculated for $a = 10^3$ and mesh size 0.1, is depicted in Supplementary Figures 3D (wt), 10D (FF) and 11D (FGS) with a dashed red line.

2.3 Ligand-receptor based Model with Equal Diffusion Coefficients, T3.

Supplementary Figures 3E (wt), 10E (FF) and 11E (FGS) shows that for the ligand-receptor based model with equal diffusion coefficients, T3, the minimum value of the deviation, Δ, decreases as the value of the parameter γ decreases. To estimate the limiting value of the deviation, Δ, at low values of γ we conducted a convergence test (Supplementary Figures 19C (wt), 20C (FF) and 21C (FGS)). The convergence test shows that the deviation, Δ attains a minimum at $\gamma = 10^{-3.5}$.

2.4 Ligand-receptor based Model with non cooperative interactions, T4.

Supplementary Figures 3F, 10F and 11F shows that for the ligand-receptor based model without cooperative interactions, T4, the minimum value of the deviation, Δ, decreases as the value of the parameter b increases. Large values of the parameter b lead to steep concentration gradients at the epithelium-mesenchyme border, which makes the computation inaccurate. To estimate the limiting value of the deviation, Δ, we ran a convergence test (Supplementary Figures 19D (wt), 20D (FF) and 21D (FGS)). The convergence test shows that the deviation, Δ, attains a minimum at $b \approx 10^{1.6}$ (wt), $b \approx 10^{2}$ (FF) and $b \approx 10^{1.6}$ (FGS).

3 Model Validation with biochemical Perturbations

Uniform addition of GDNF ligand to the embryonic kidney culture results in a widening of the buds (Supplementary Figure 14A,B). This effect can be qualitatively recapitulated *in silico* (Supplementary Figure 14C,D), and is a consequence of a saturation of signalling-dependent tissue growth at receptor-saturating GDNF concentrations. We next studied the effect of a locally restricted GDNF ligand source in the form of a GDNF-loaded bead (Supplementary Figures 15, 16). The effect of a local source of ligand, in this case GDNF, on ligand-receptor based Turing patterns can be understood by recalling that the instability that arises from the ligand-receptor interactions has a particular wave-length, that manifests itself as the distance between branching points. This wavelength is dependent on the parameter values, including the ligand production and turn-over rates. A local source of ligand (GDNF) enhances the local availability of the ligand and therefore modulates the wavelength. Simulations on a simplified, static two-layer domain, that idealises the epithelial and mesenchymal layers, show that at a sufficiently high concentration of the ligand in the bead, an additional spot with high ligand-receptor signalling can emerge either directly near the local ligand source (Supplementary Figure 15A-C), or a peak can split into two peaks (Supplementary Figure 15D-F). The exact effect of an additional ligand source is difficult to anticipate due to the nonlinear nature of the system and must therefore be studied computationally. GDNF-loaded beads have both of the above described effects in growing embryonic kidney cultures: a) a widening of the epithelial tissue in the proximity of the bead due to saturation of growth, and b) increased branching and growth toward the bead (Supplementary Figure 16A,B). The simulations recapitulate the observed behaviour qualitatively (Supplementary Figure 16C,D). The effect of the GDNF-loaded bead was confirmed by its effect on *Etv*4 expression. The GDNF-loaded bead indeed increased *Etv*4 expression levels in the epithelial tips in proximity of the GDNF source (Supplementary Figure 2), therefore pointing to a local increase in RET signalling.

Supplementary Methods

1 Analysis of Interbud Distances in 3D Images

To quantify the interbud distance in 3D images of wild type and *Wnt11* mutant kidneys, we implemented the following image analysis pipeline in Fiji 2.0 [3] and MATLAB 8.4 (The MathWorks Inc., Natick, MA, 2014).

OPT Image Processing

1. The median filter [4] was applied to reduce noise in reconstructed OPT images.
2. Filtered images were segmented with the local Otsu filter [5] implemented in the Fiji Auto Local Threshold plugin collection [6]; the value for the radius of the local filter was set to 50 voxels.
3. Segmented images were further cleaned by filling voids in the segmentation and by removing objects comprising only a small number of voxels. The segmentation of a representative kidney epithelium is depicted in Supplementary Figure 22A.
4. Binary images were skeletonized with the Skeletonize3D [7] Fiji plugin (Supplementary Figure 22B).
5. Voxels of skeletonized images were classified as slab, junction, or end-point with the AnalyzeSkeleton [8] Fiji plugin. Classified skeletons were exported to MATLAB for further analysis of interbud-distances (Supplementary Figure 22C);

Interbud Distance Analysis

Visual inspection of kidney images suggests that buds with minimal bud to bud distance are typically branched off from the same branching point (node) (Supplementary Figure 22D) and therefore reflect the distance buds have grown away from each other, rather than the minimal distance to which buds can approach each other. Given that bifurcations and trifurcations prevail during kidney branching morphogenesis [9], the third and further (Supplementary Figure 22D) shortest distances should represent an adequate measure for the distance by which buds can approach each other. Supplementary Figure 22E, F depicts the distribution and median distance to n-th buds. In an alternative approach the density of ureteric buds was estimated as the ratio of the kidney surface area to the number of epithelial tips.

The kidney surface area was approximated by the area of an ellipsoid that was fitted (*ellipsoid_fit* [10] MATLAB function) to the coordinates of epithelial tips.

Supplementary Tables

Supplementary Table-1. A summary of the evaluated models. E and M stands for the epithelium and for the mesenchyme, accordingly.

	Diagram	Equations	Description
T1	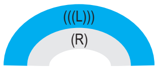	$E: \begin{cases} R_t = \Delta R + \gamma(a - R + R^2 L) \\ L_t = D\Delta L + \gamma(-R^2 L) \end{cases}$ $M: \; L_t = D\Delta L + \gamma(b - L)$	R is expressed and diffuses in the epithelium. L is expressed in the mesenchyme, diffuses everywhere and binds to R in the epithelium.
T2	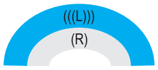	$E: \begin{cases} R_t = \Delta R + \gamma(a - R - R^2 L) \\ L_t = D\Delta L + \gamma(-R^2 L) \end{cases}$ $M: \; L_t = D\Delta L + \gamma(b - L)$	R is expressed and diffuses in the epithelium. L is expressed in the mesenchyme, diffuses everywhere and binds to R in the epithelium.
T3	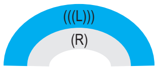	$E: \begin{cases} R_t = \Delta R + \gamma(a - R + R^2 L) \\ L_t = \Delta L + \gamma(-R^2 L) \end{cases}$ $M: \; L_t = \Delta L + \gamma(b - L)$	R is expressed and diffuses in the epithelium. L is expressed in the mesenchyme, diffuses everywhere and binds to R in the epithelium.
T4	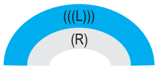	$E: \begin{cases} R_t = \Delta R + \gamma(a - R + RL) \\ L_t = \Delta L + \gamma(-RL) \end{cases}$ $M: \; L_t = \Delta L + \gamma(b - L)$	R is expressed and diffuses in the epithelium. L is expressed in the mesenchyme, diffuses everywhere and binds to R in the epithelium.
T5	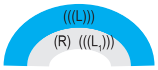	$E: \begin{cases} R_t = \Delta R + \gamma(a_0 + R^2 L - R) \\ L_t = D\Delta L + \gamma(-R^2 L) \\ L_{1t} = D\Delta L_1 + \gamma(\rho_0 + \rho_1 \frac{R^2 L}{1+R^2 L} - \delta L_1) \end{cases}$ $M: \begin{cases} L_t = D\Delta L + \gamma(b_0 + b_1 \frac{L_1^2}{1+L_1^2} - L) \\ L_{1t} = D\Delta L + \gamma(-\delta L_1) \end{cases}$	R is expressed and diffuses in the epithelium. L is expressed in the mesenchyme, diffuses everywhere and binds to R in the epithelium. L_1 is expressed in the epithelium, diffuses everywhere and increases L expression in the mesenchyme.

Supplementary Table-2. *In Silico* Branching Morphogenesis: Parameters and Initial Geometries. $D = 100$ in all models.

Type	Initial Geom	γ	a	b	growth function
wt	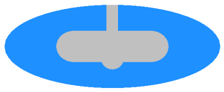	0.03	0.8	1.44	$\frac{R^2L}{\max(R^2L)}$
FF	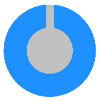	0.1	0.8	1	$\frac{R^2L}{\max(R^2L)}$
FGS	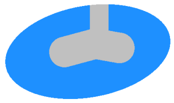	0.03	2	0.4	$\frac{R^2L}{\max(R^2L)}$
wt+GDNF[1]	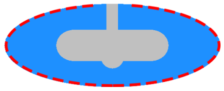	0.03	0.8	1.44	$\frac{(R^2L)^n}{K^n+(R^2L)^n}$
wt+bead[1]	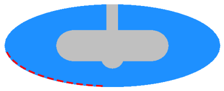	0.03	0.8	1.44	$\frac{(R^2L)^n}{K^n+(R^2L)^n}$
T1[2]	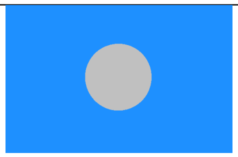	0.02	2	0.8	$\frac{R^2L}{K+R^2L}$
T5[3]	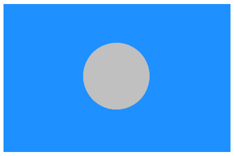	0.02	0.2	0.5	$\frac{R^2L}{K+R^2L}$

[1] Dotted red line indicates source of GDNF at the outer mesenchymal boundary. n = 2, K = 20.

[2] The mesenchyme in the model was virtually infinitely large, so that a further increase in the size of the mesenchyme did not affect the computational result.

[3] The mesenchyme in the model was virtually infinitely large, so that an increase in the size of the mesenchyme did not affect the computational result. Values of the other parameters: $b_1 = 18$, $\rho_0 = 0$, $\rho_1 = 1$, $\delta = 10$, $K = 0.6 * \max(R^2 L)$.

Supplementary Figures

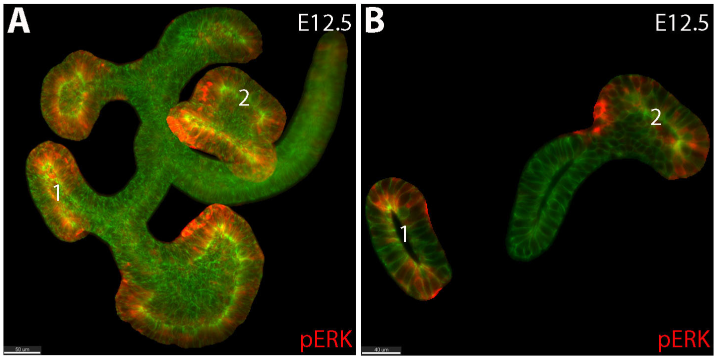

Supplementary Figure 1. Phoshorylated ERK is concentrated in the UB Epithelial Tips. (A) A maximum intensity projection of an E12.5 HoxB7Venus kidney (green) stained with an antibody against pERK (red); (B) a close up of a cross-section of epithelial buds 1 and 2, depicted in panel (A).

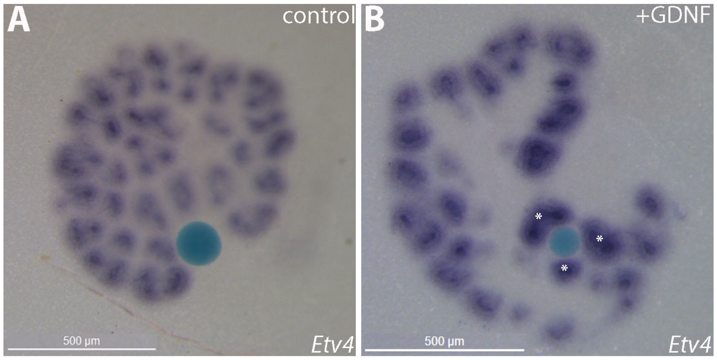

Supplementary Figure 2. A local GDNF source enhances *Etv4* expression levels. *Etv4* expression levels in E11.5 kidney explants that have been cultured for 48 hours either with (A) control, PBS soaked bead, or (B) in the presence of a GDNF soaked bead (10 ng/ml). Asterisks in (B) point to enlarged buds with higher *Etv4* expression near the GDNF soaked bead.

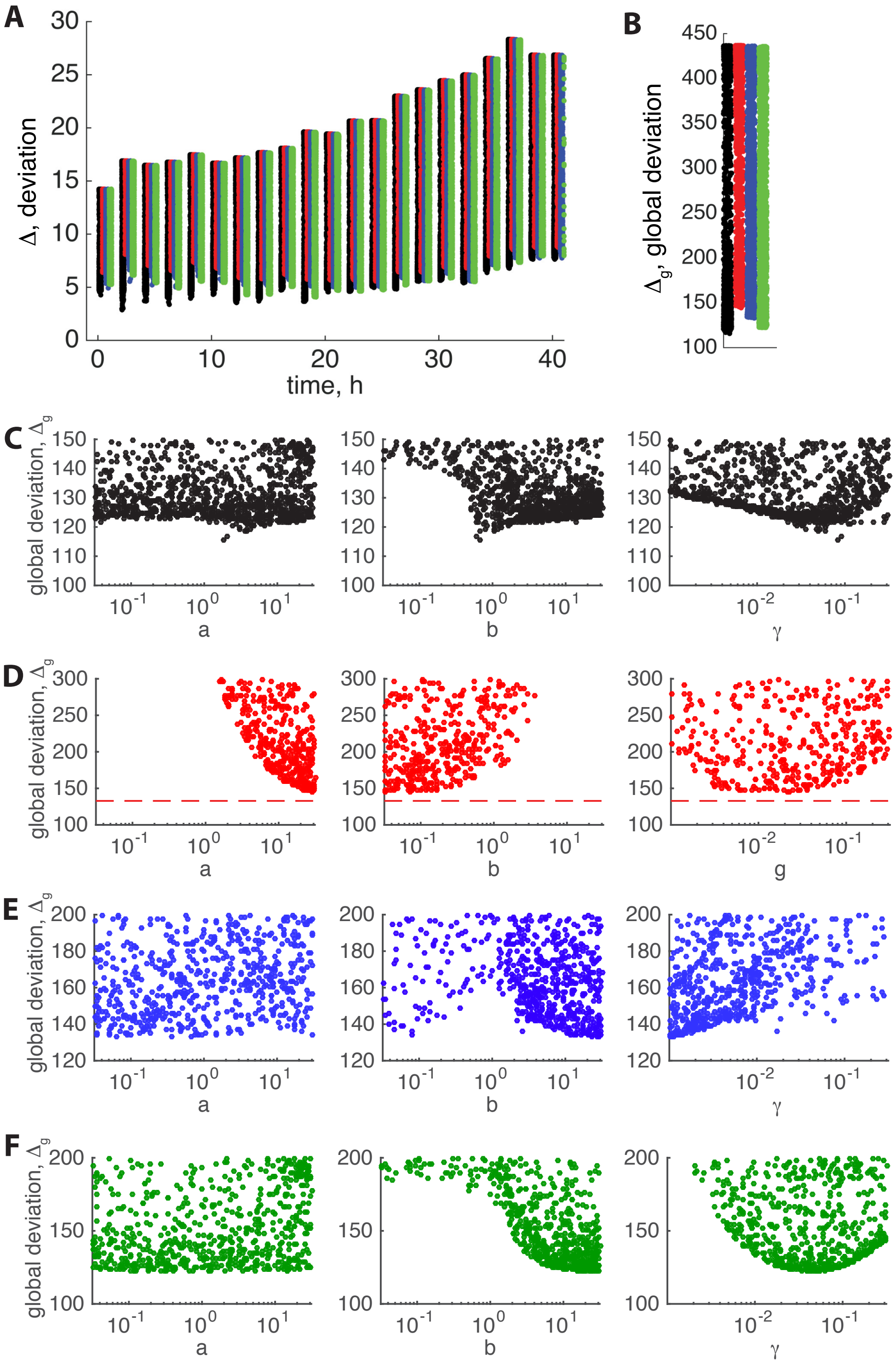

Supplementary Figure 3. Parameter Screens for all four Models T1-T4 for wild type kidney data. The full caption is on the next page.

Supplementary Figure 3. Parameter Screens for all four Models T1-T4 for wild type kidney data. (A) The deviation Δ (Eq 4), between the spatial distribution of the predicted signalling strength C and the experimentally determined growth field, E. Each coloured dot represents the deviation Δ for a specific model and parameter set at the specified time point. Black - the complete ligand-receptor based model (Table Supplementary Table-1: T1), red - a model without receptor up-regulation (Table Supplementary Table-1: T2), green - a model where receptor and ligand diffusion coefficients were set equal (Table Supplementary Table-1: T3), and blue - a model with 1:1 stoichiometry of the ligand-receptor complex (Table Supplementary Table-1: T4). (B) Global deviation, Δ_g (Eq 5) by summing over all time points. The colour code is identical to that in panel A. (C-F) The global deviation, Δ_g (Eq 5) versus the specified parameter values calculated for (C) the complete ligand-receptor based model (T1), (D) a model without receptor up-regulation (T2); the red dashed line depicts the limiting value of deviation, Δ obtained for high values of a, (E) a model where receptor and ligand diffusion coefficients were set equal (T3), (F) a model with 1:1 stoichiometry of the ligand-receptor complex (T4).

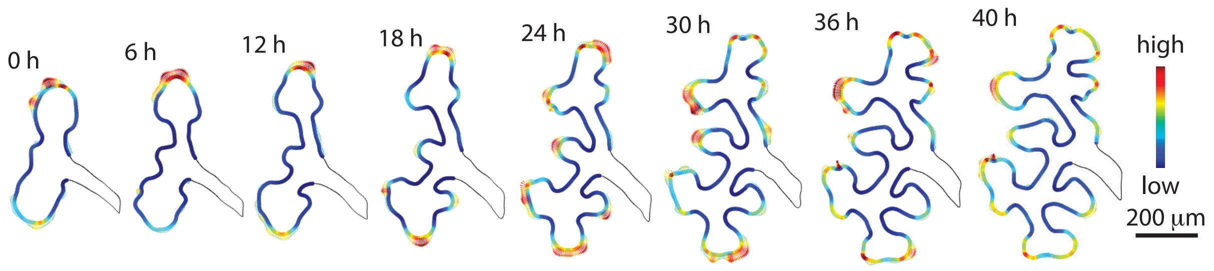

Supplementary Figure 4. Image-based Data from wild type kidneys supports a ligand-receptor based Turing Mechanism. The growth areas predicted by the ligand-receptor based model (solid colour) match the growth fields extracted from the experimental data (vectors). Optimal parameter values were chosen for every time frame.

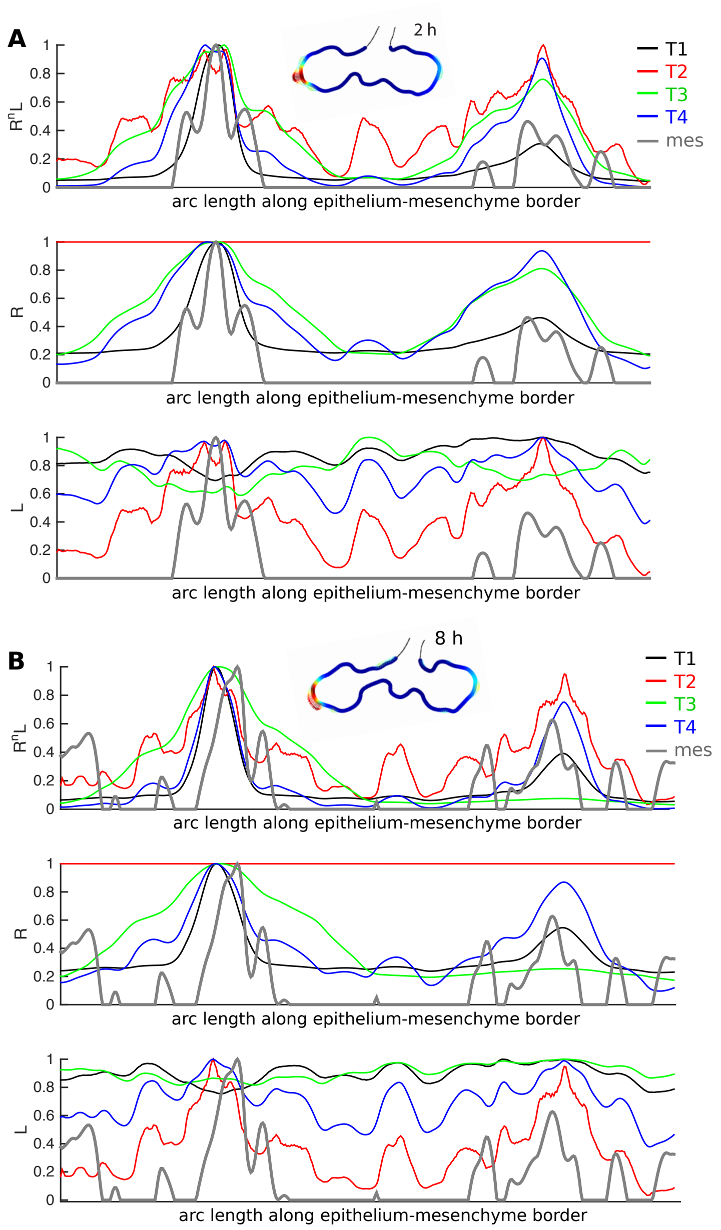

Supplementary Figure 5. Comparison of measured and predicted Growth Areas. Comparison of growth areas as extracted from the imaging data (in grey) and the concentrations of (top row) the ligand-receptor complex, (center row) the receptor, and (bottom) the ligand as predicted by the complete ligand-receptor based model (Table Supplementary Table-1: T1, in black), a model without receptor up-regulation (Table Supplementary Table-1: T2, in red), a model where receptor and ligand diffusion coefficients were set equal (Table Supplementary Table-1: T3, in green) and a model with 1:1 stoichiometry of the ligand-receptor complex (Table Supplementary Table-1: T4, in blue). Panels (A) and (B) correspond to the time frames at 2 and 8 hours. The corresponding ureteric bud shape with the calculated displacement field (vectors) and the best matching signalling field (solid colours) is shown as inset.

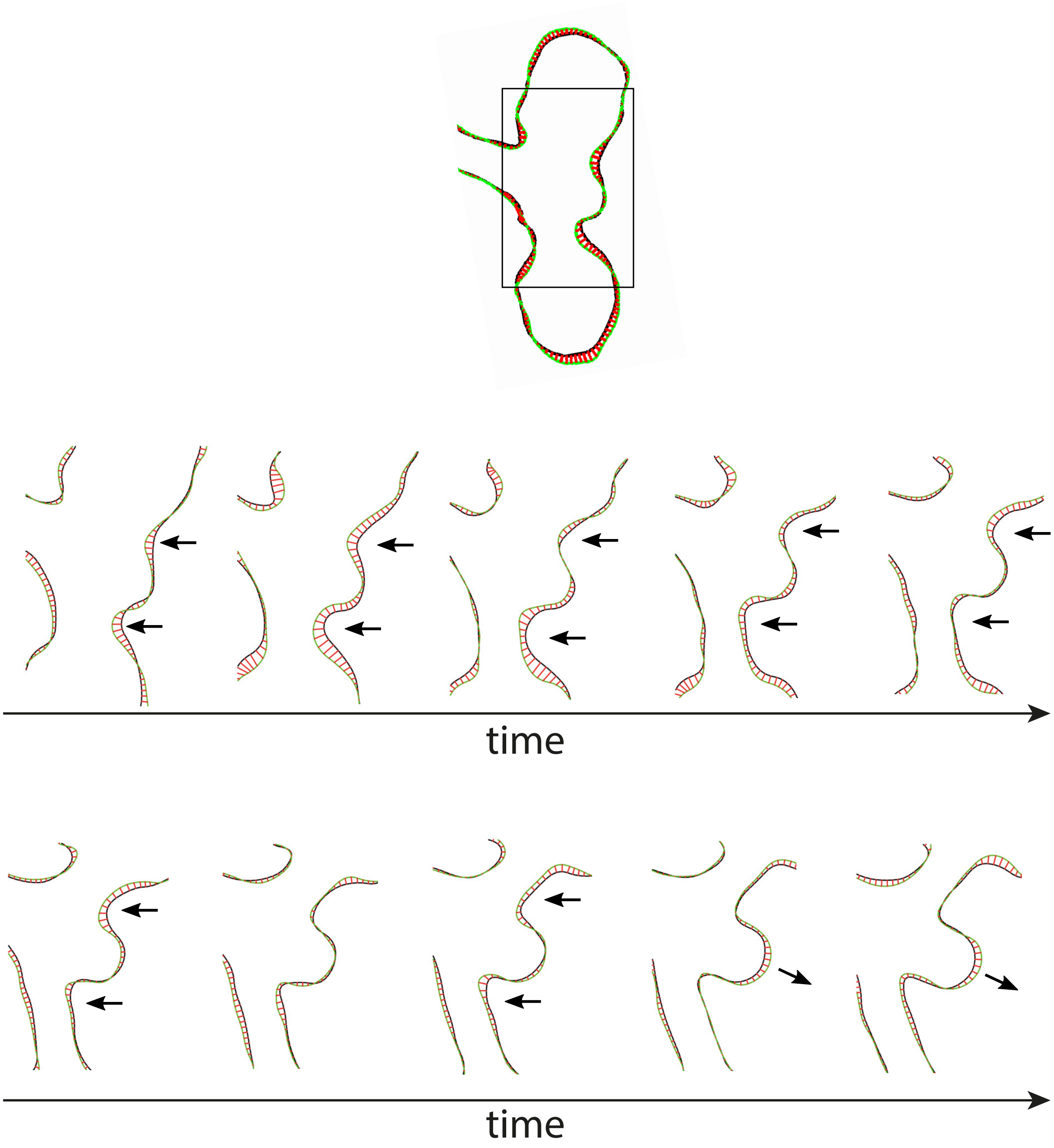

Supplementary Figure 6. Close-up of the Central Bud. The displacement field (red arrows) between the epithelial border of an earlier (black line) and a later (green line) time point in two consecutive frames shows that the central bud initially emerges primarily due to a shrinkage of the surrounding epithelial tissue and only at the later times due to the epithelium protrusion. Black arrows indicate predominant local direction of the epithelial border movement.

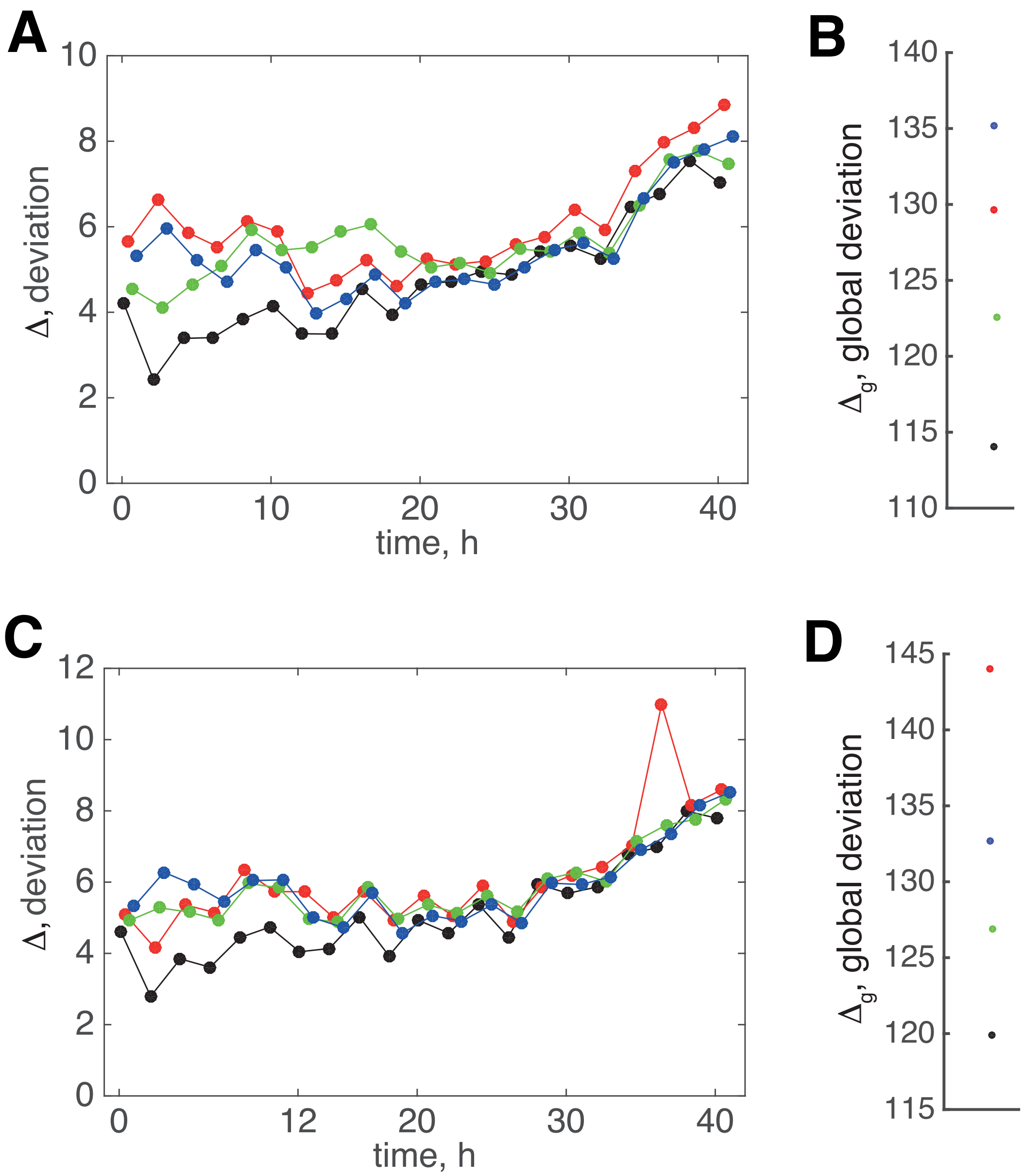

Supplementary Figure 7. Image-based Data from wild type kidneys supports a ligand-receptor based Turing Mechanism: The Impact of the Mesenchyme Size. Comparison of (A, C) the minimum deviation, Δ (Eq 4), of the spatial distribution of signalling strength, C, from the growth field, E, obtained form the experimental data, and (B, D) the minimum of the global deviation Δ_g for the entire time course of kidney branching morphogenesis for a mesenchyme with ellipse size $\alpha = 4.5$ (top row) and a mesenchyme with ellipse size $\alpha = 2.5$ (bottom row). In both cases, the complete ligand-receptor based model (Table Supplementary Table-1: T1, black), performs better than any of the non-Turing mechanisms (red - a model without receptor up-regulation (Table Supplementary Table-1: T2), green - a model where receptor and ligand diffusion coefficients were set equal (Table Supplementary Table-1: T3), and blue - model with 1:1 stoichiometry of the ligand-receptor complex (Table Supplementary Table-1: T4)).

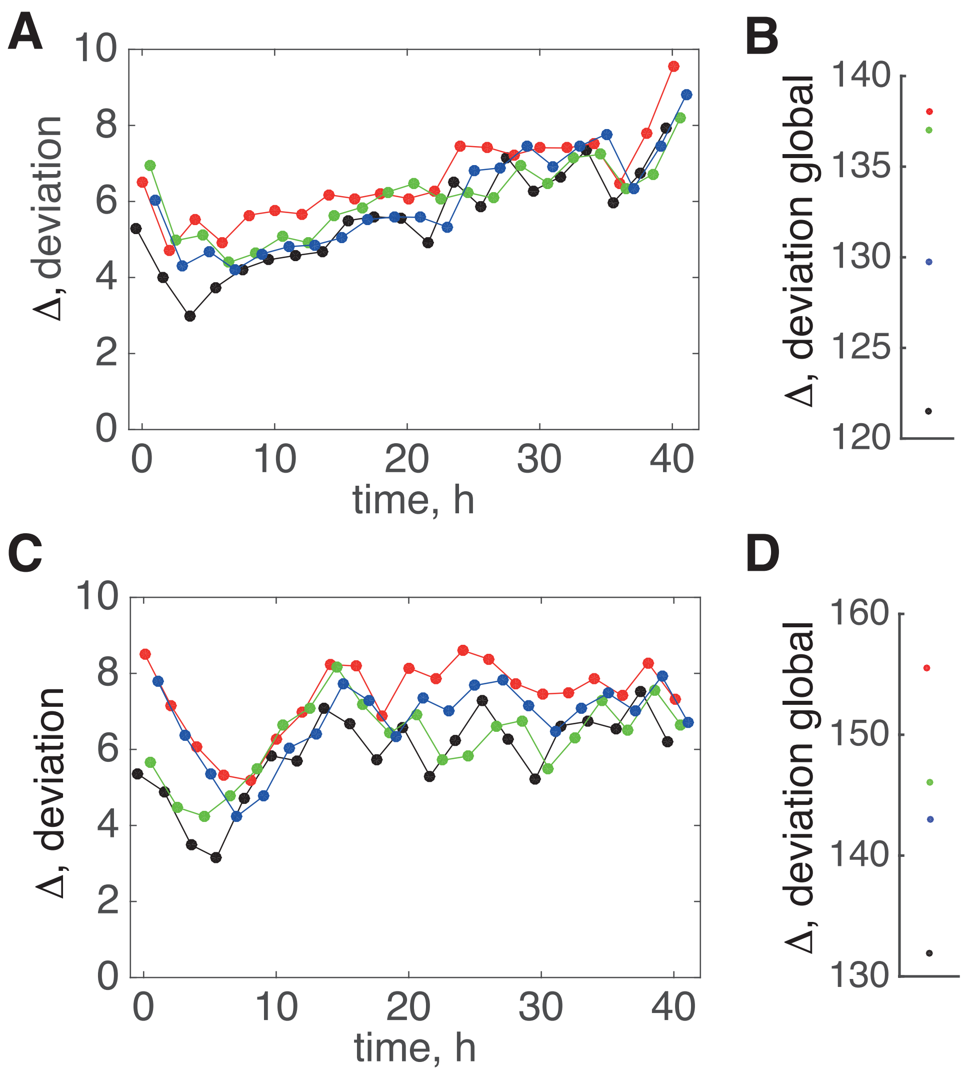

Supplementary Figure 8. Image-based Data from wild type kidneys supports a Ligand-receptor based Turing Mechanism: Analysis of additional independent datasets. (A, C) The minimum deviation, Δ (Eq 4), of the spatial distribution of signalling strength, C, from the growth field, E, obtained form the experimental data, and (B, D) the minimum of the global deviation Δ_g for the entire time course of kidney branching morphogenesis for two further independent datasets (top and bottom rows). Panels A, B and C, D depict analysis of two independent experiments. In all cases, the complete ligand-receptor based model (Table Supplementary Table-1: T1, black), performs better than any of the non-Turing mechanisms (red - a model without receptor up-regulation (Table Supplementary Table-1: T2), green - a model where receptor and ligand diffusion coefficients were set equal (Table Supplementary Table-1: T3), and blue - model with 1:1 stoichiometry of the ligand-receptor complex (Table Supplementary Table-1: T4)).

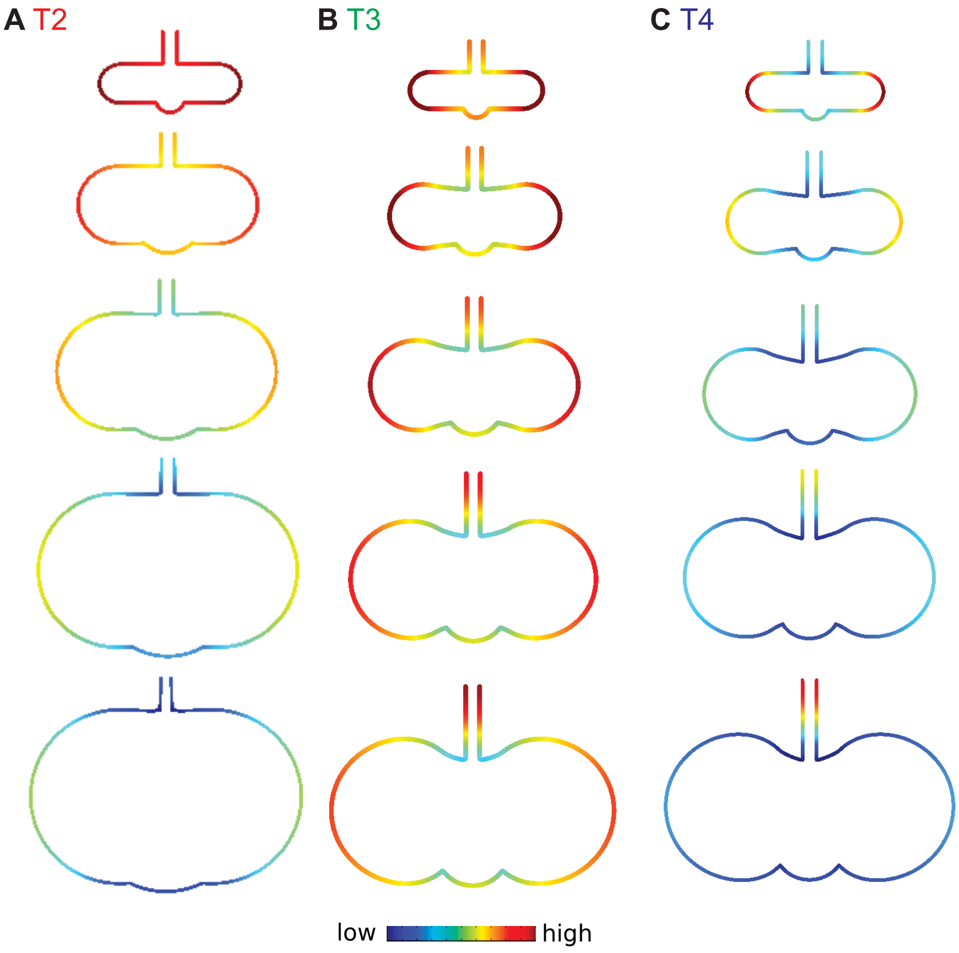

Supplementary Figure 9. ***In Silico*** **Morphogenesis, wild type Kidney.** The non-Turing ligand-receptor based models (A) without positive feedback, T2, (B) without cooperative interactions, T3, and (C) with equal diffusion coefficients, T4, fail to induce branching morphogenesis. The initial shape of the computational domain qualitatively resembles that of wild type kidney explants and is the same as in the simulation for the ligand-receptor based model, T1 (Fig 3G). The model parameters are summarised in Table Supplementary Table-2. Blue and red indicate low and high levels of ligand-receptor signalling, accordingly.

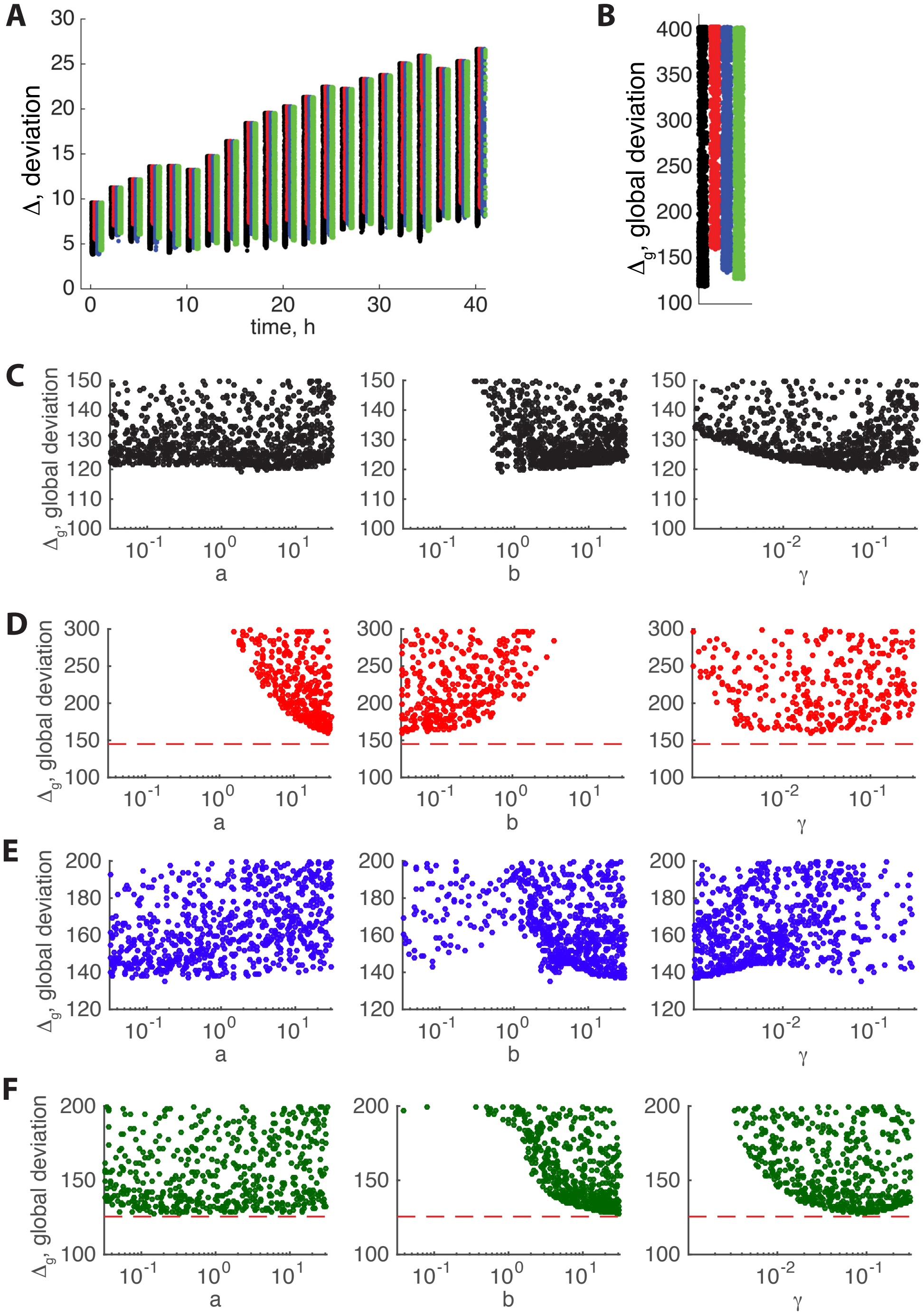

Supplementary Figure 10. Parameter Space of the Ligand-receptor based Models: FF Mutant. The full caption is on the next page.

Figure 10. Parameter Screens for all four Models T1-T4 for data of the FF mutant kidney. (A) The deviation Δ (Eq 4), of the spatial distribution of signalling strength C from the growth field, E, extracted from the experimental data. Each coloured dot represents a deviation Δ, which has been calculated for a specific parameter set and at a given time. Black - the complete ligand-receptor based model (Table Supplementary Table-1: T1), red - a model without receptor up-regulation (Table Supplementary Table-1: T2), green - a model where receptor and ligand diffusion coefficients were set equal (Table Supplementary Table-1: T3), and blue - a model with 1:1 stoichiometry of the ligand-receptor complex (Table Supplementary Table-1: T4). (B) Global deviation, Δ_g (Eq 5). The colour code is identical to that in panel A. (C-F) The global deviation, Δ_g (Eq 5) versus the parameter values calculated for (C) the complete ligand-receptor based model (T1), (D) a model without receptor up-regulation (T2); the red dashed line depicts the limiting value of deviation, Δ obtained for high values of a, (E) a model where receptor and ligand diffusion coefficients were set equal (T3), (F) a model with 1:1 stoichiometry of the ligand-receptor complex (T4).

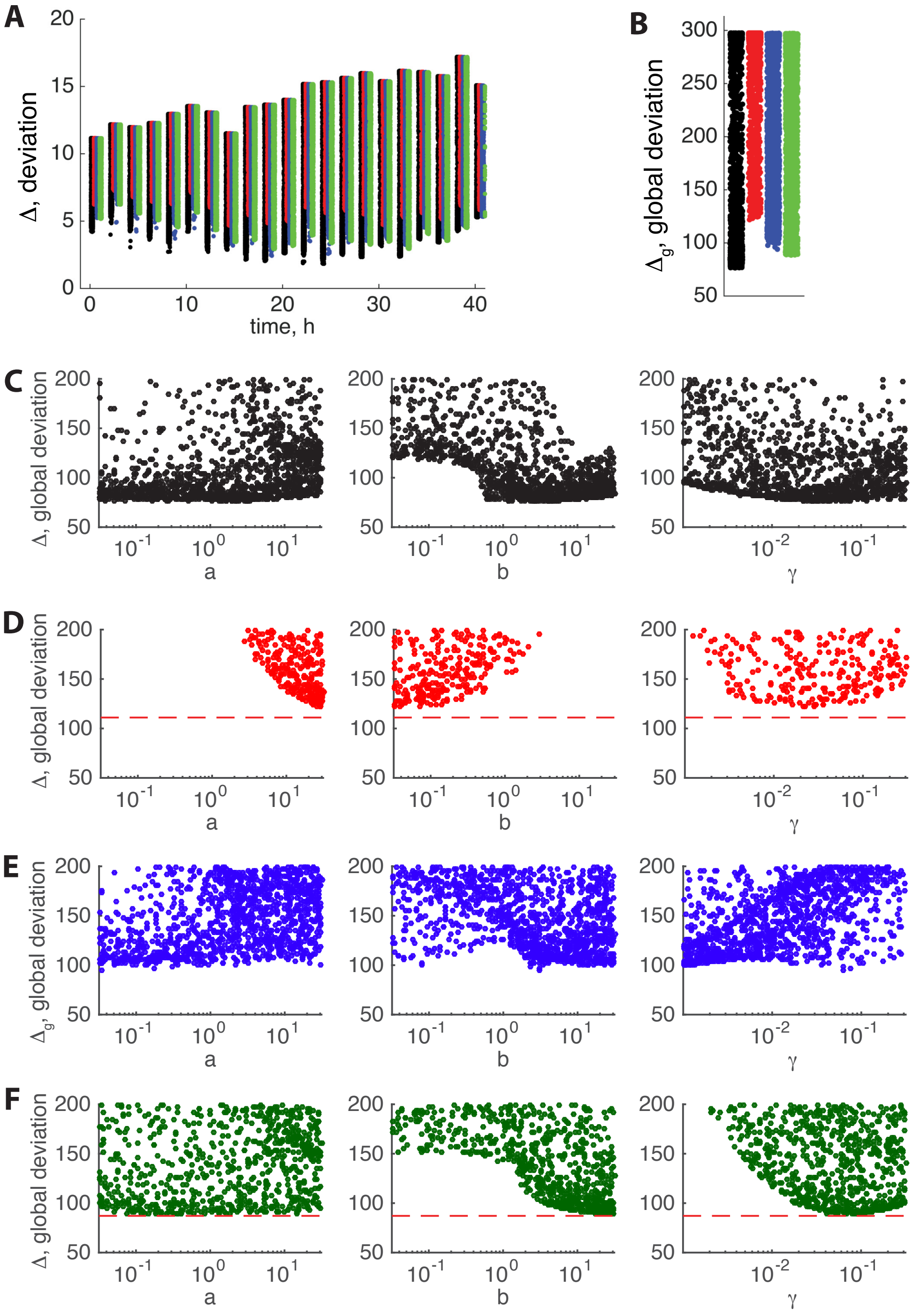

Supplementary Figure 11. Parameter Space of the Ligand-receptor based Models: FGS Mutant. The full caption is on the next page.

Figure 11. Parameter Screens for all four Models T1-T4 for data of the FGS mutant kidney. (A) The deviation Δ (Eq 4), of the spatial distribution of signalling strength C from the growth field, E, extracted from the experimental data. Each coloured dot represents a deviation Δ, which has been calculated for a specific parameter set and at a given time. Black - the complete ligand-receptor based model (Table Supplementary Table-1: T1), red - a model without receptor up-regulation (Table Supplementary Table-1: T2), green - a model where receptor and ligand diffusion coefficients were set equal (Table Supplementary Table-1: T3), and blue - a model with 1:1 stoichiometry of the ligand-receptor complex (Table Supplementary Table-1: T4). (B) Global deviation, Δ_g (Eq 5). The colour code is identical to that in panel A. (C-F) The global deviation, Δ_g (Eq 5) versus the parameter values calculated for (C) the complete ligand-receptor based model (T1), (D) a model without receptor up-regulation (T2); the red dashed line depicts the limiting value of deviation, Δ obtained for high values of a, (E) a model where receptor and ligand diffusion coefficients were set equal (T3), (F) a model with 1:1 stoichiometry of the ligand-receptor complex (T4).

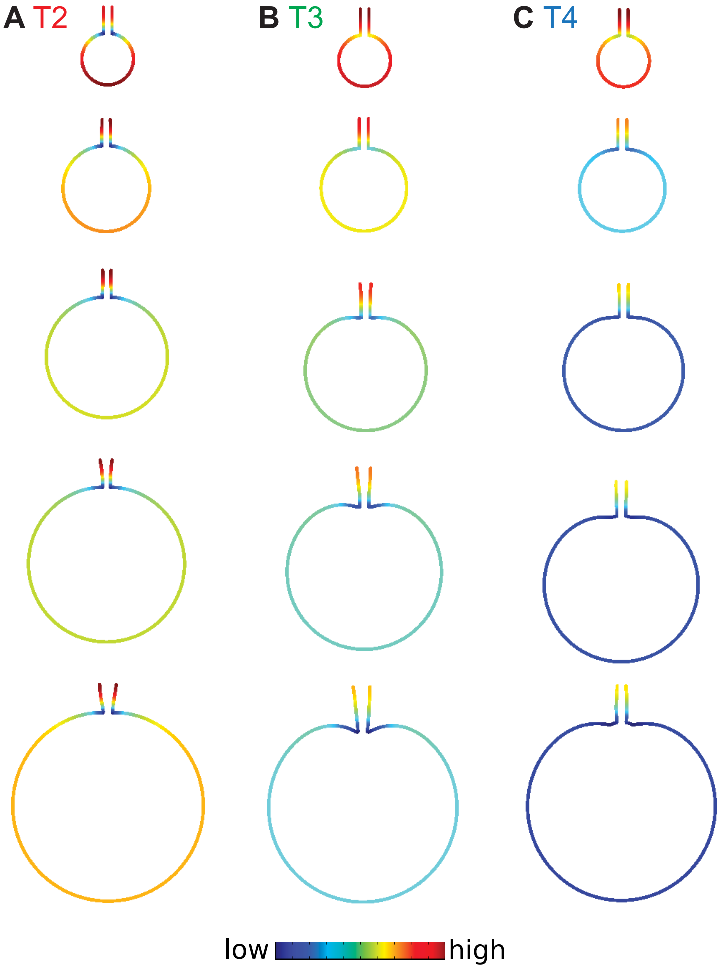

Supplementary Figure 12. ***In Silico* Morphogenesis, FF Kidney.** The non-Turing ligand-receptor based models (A) without positive feedback, T2, (B) without cooperative interactions, T3, and (C) with equal diffusional coefficients, T4, fail to induce branching morphogenesis. The initial shape of the computational domain qualitatively resembles that of FF ($Fgf10^{-/-}$) kidney explants and is the same as in the simulation for the ligand-receptor based model, T1 (Fig 3G). The model parameters are summarised in Table Supplementary Table-2. Blue and red indicate low and high levels of ligand-receptor signalling, accordingly.

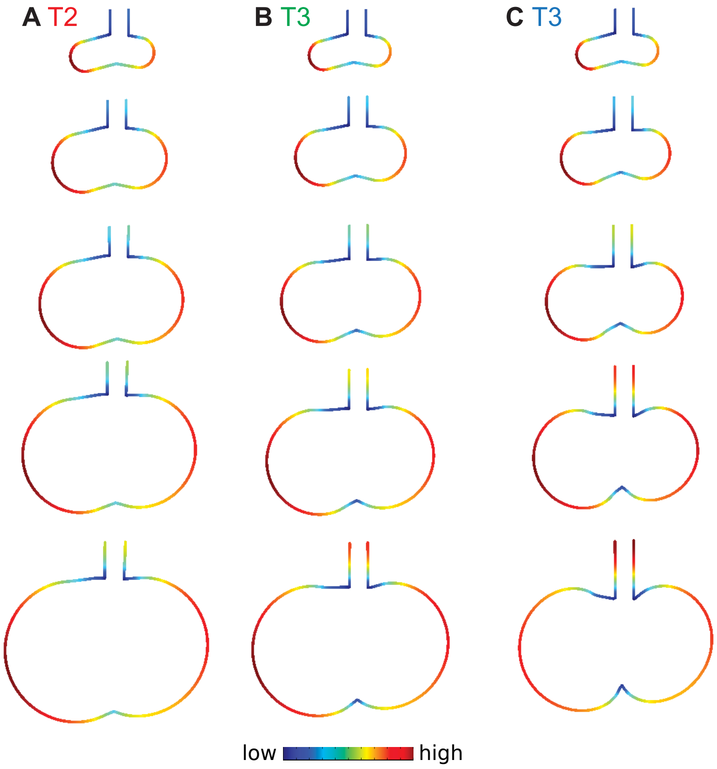

Supplementary Figure 13. ***In Silico*** **Morphogenesis, FGS Kidney.** The non-Turing ligand-receptor based models (A) without positive feedback, T2, (B) without cooperative interactions, T3, and (C) with equal diffusional coefficients, T4, fail to induce branching morphogenesis. The initial shape of the computational domain qualitatively resembles that of FGS ($FGF10^{+/-}$;$Gdnf^{+/-}$;$Spry1^{+/-}$) kidney explants and is the same as in the simulation for the ligand-receptor based model, T1 (Fig 3H). The model parameters are summarised in Table Supplementary Table-2. Blue and red indicate low and high levels of ligand-receptor signalling, accordingly.

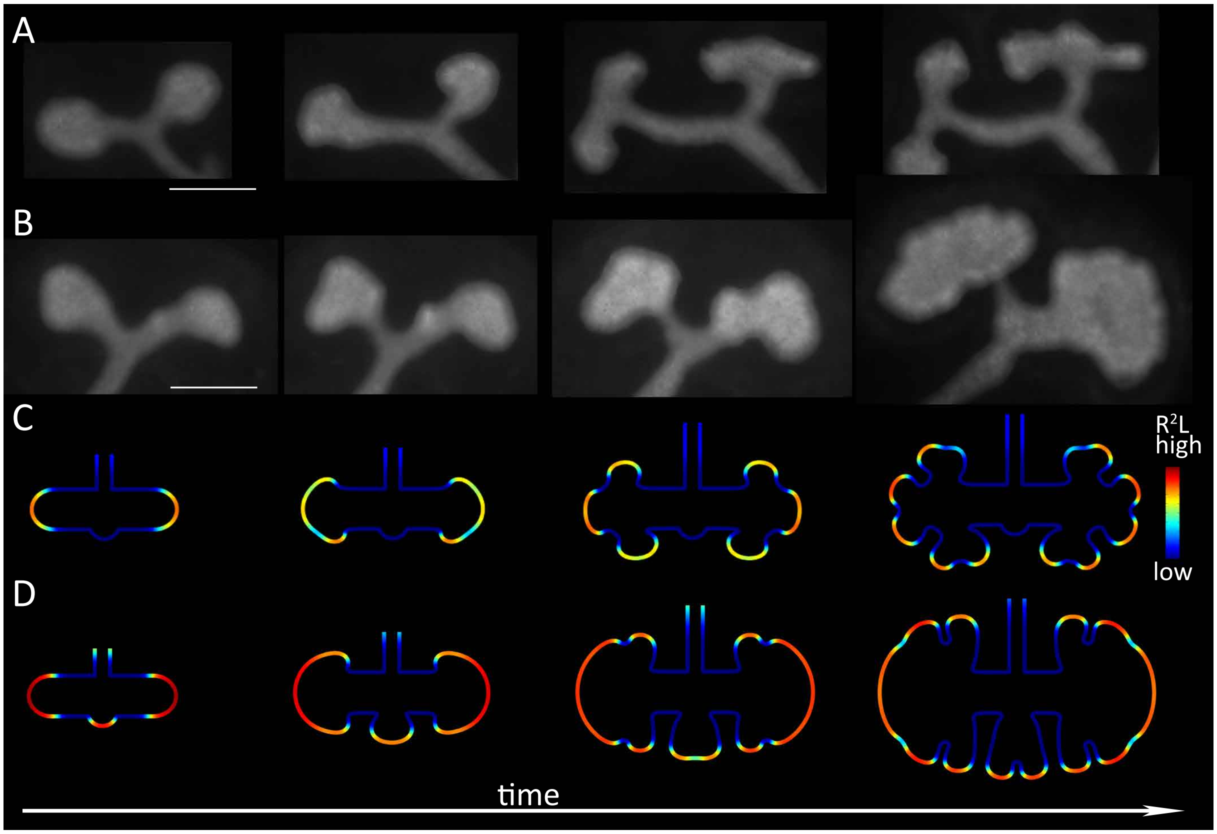

Supplementary Figure 14. Biochemical Perturbation of embryonic kidney explant cultures with uniformly added GDNF. (A,B) Snapshots from the 24 hours time-lapse movie of kidney branching morphogenesis: (A) control, (B) in presence of uniformly added GDNF (100ng/ml). The scale bar corresponds to 200 μm. (C,D) *In Silico* branching morphogenesis: (C) control, (D) in the presence of externally added GDNF. The colour code indicates the concentration of the ligand-receptor complex, R^2L, on the epithelial domain. The domain growth in normal direction to the epithelial layer; the velocity field is dependent on the ligand-receptor complex, R^2L. The model parameters are summarised in Table Supplementary Table-2.

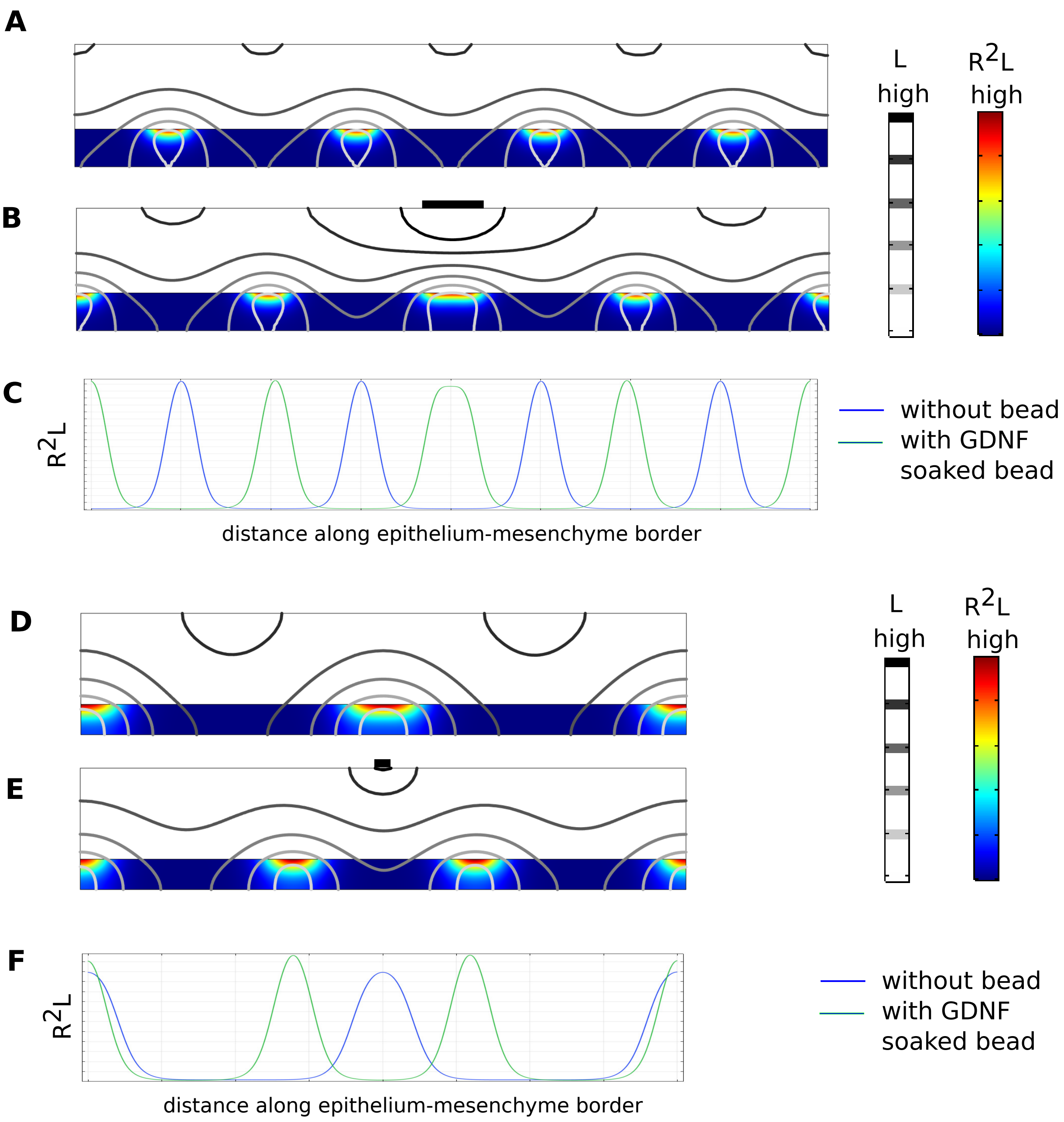

Supplementary Figure 15. Effect of a Local Ligand Source on the patterns generated by a ligand-receptor based Turing-type Mechanism. Panels A-C (a=0.4, b=1, $\gamma = 0.05$, D=100) and panels D-F (a=0.4, b=1, $\gamma = 0.02$, D=100) were simulated, as indicated in the brackets, with two different parameterisations of the Eq Supplementary Equation-10 describing ligand-receptor based interactions. (A, D) Simulated ligand-receptor-based Turing pattern on a simple two-layered rectangular domain. The top layer represents the ligand-producing mesenchyme, while the lower layer represents the receptor-expressing epithelium. (B, E) Simulated ligand-receptor-based Turing model in the presence of a local source of GDNF, as indicated with a black rectangular at the top of the epithelial domain. (C, F) The concentration of the ligand-receptor complex, R^2L, along the interface of the two domains.

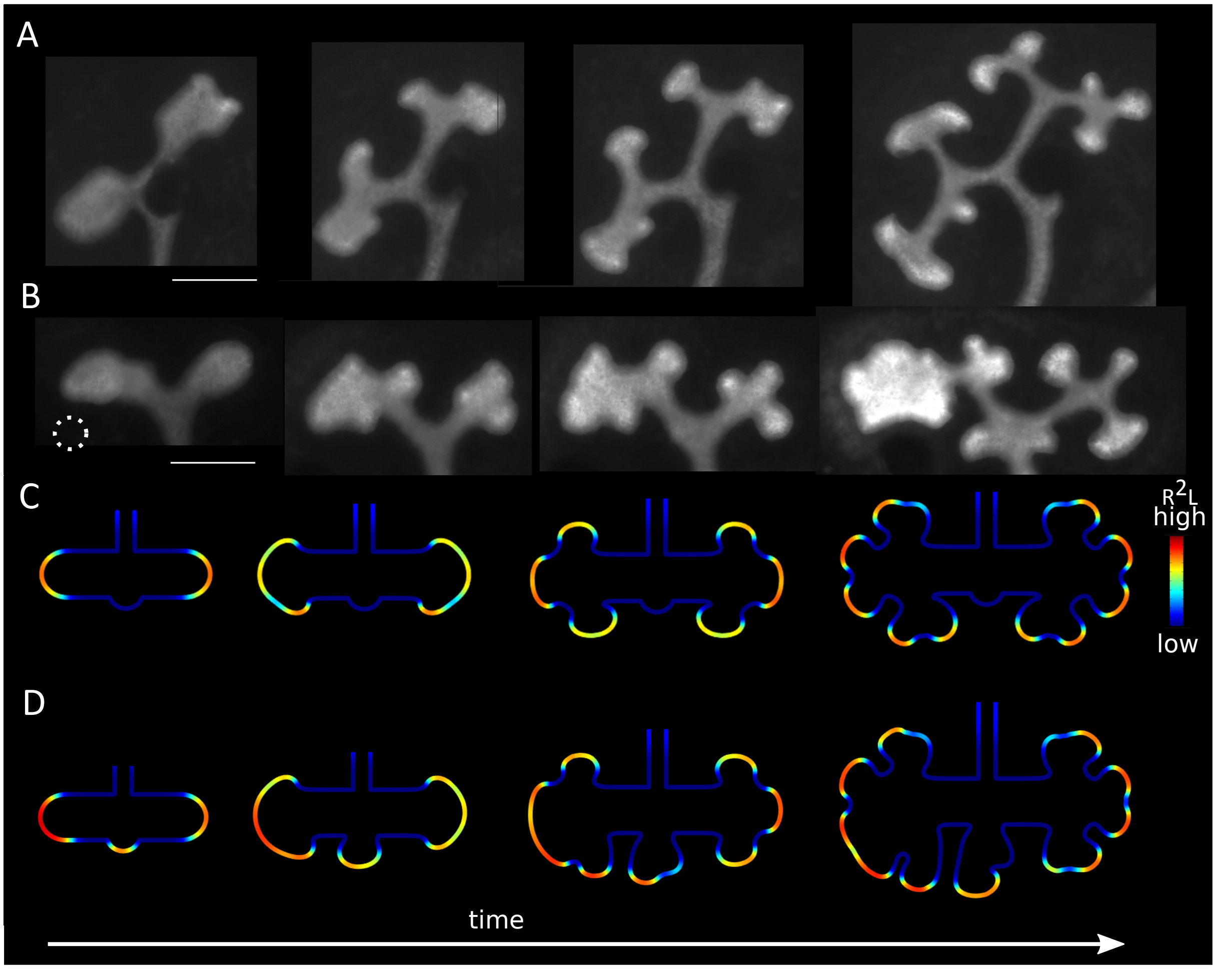

Supplementary Figure 16. Biochemical Perturbation of embryonic kidney explant cultures with a local source of GDNF. (A,B) Snapshots from the 24 hours time-lapse movie of kidney branching morphogenesis: (A) control, PBS soaked bead (B) in the presence of a GDNF-soaked bead (10 ng/ml). The bead position is indicated schematically with a dashed circle. The scale bar corresponds to 200 μm. (C,D) *In Silico* branching morphogenesis on an idealised domain: (C) control, (D) in presence of local GDNF source. The colour code indicates the concentration of the ligand-receptor complex, R^2L, on the epithelial domain. The domain growth in normal direction to the epithelial layer; the velocity field is dependent on the ligand-receptor complex, R^2L. The model parameters are summarised in Table Supplementary Table-2.

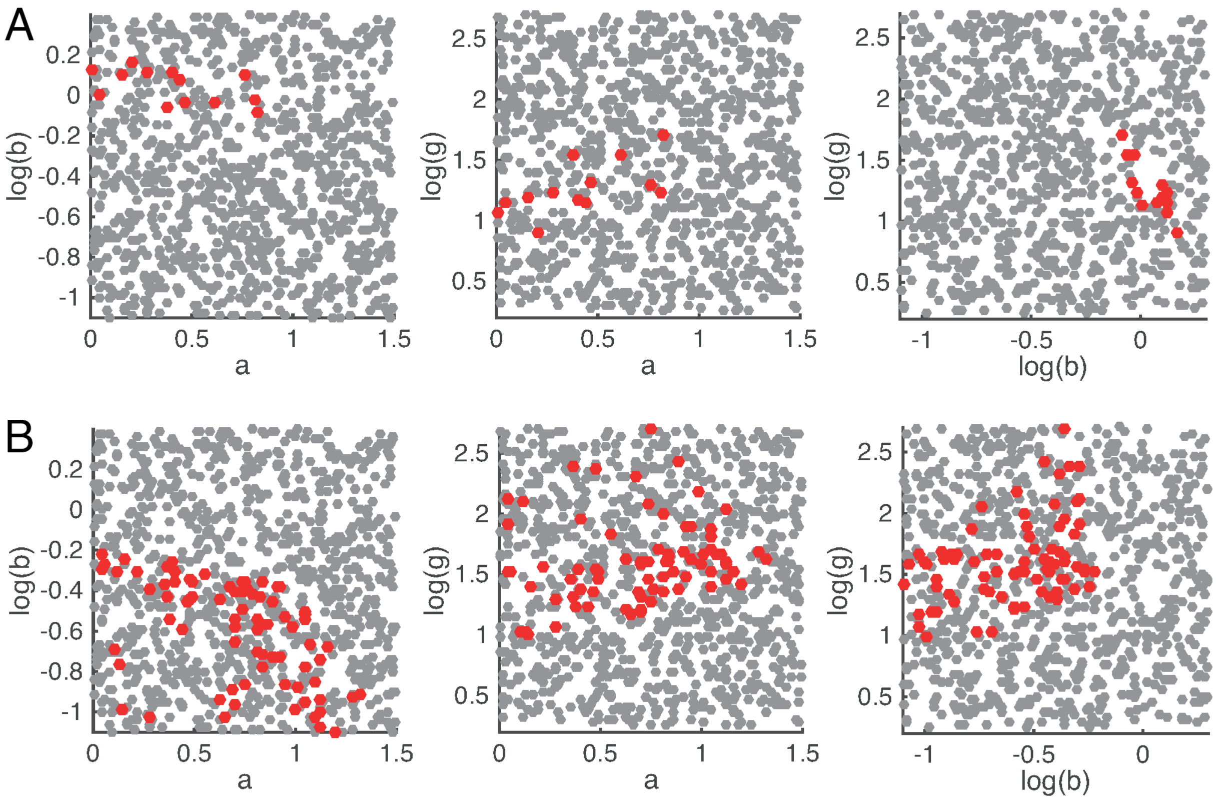

Supplementary Figure 17. Parameter space for T1 and T5 models. Projection of the parameter space for the complete ligand-receptor based Turing model T1 (A) and the one with an additional positive feedback T5 (B). The red dots depict parameter sets, which lead to the elongation mode of branching. The grey dots depict parameters, which lead to any other pattern.

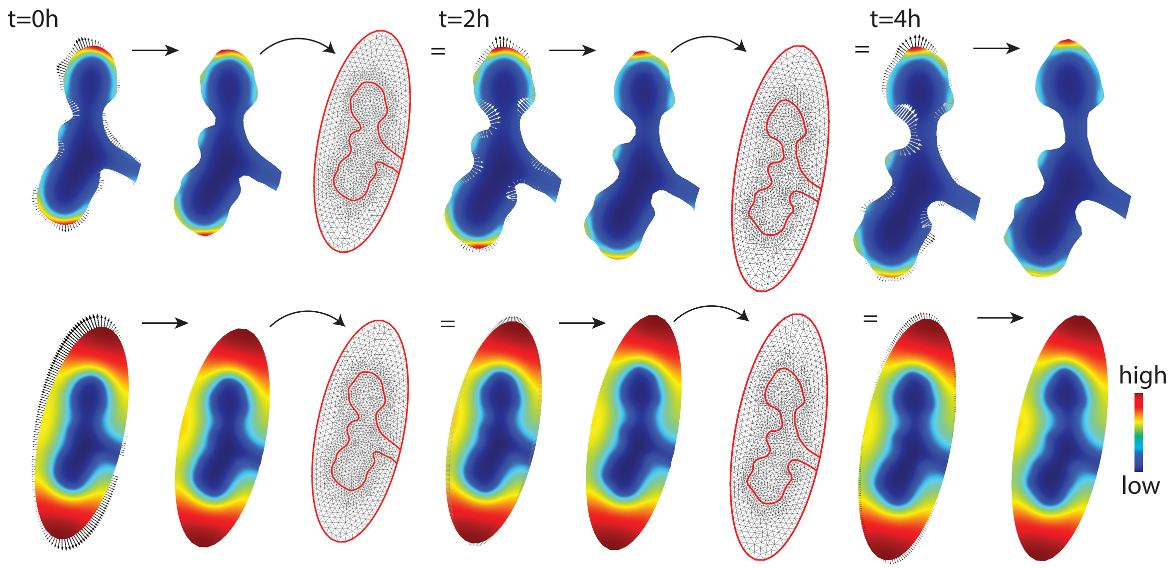

Supplementary Figure 18. Numerical Computations: Growing Domain, Prescribed Growth. Solving the ligand-receptor based Turing-type model on a growing domain. The upper and lower rows depict the concentration of the receptor and the ligand, accordingly. The vector fields indicate the displacement fields, the wireframe rendering represents the computational FEM mesh; note that the mesh used to carry out the computations reported in the manuscript was finer than the one depicted here, but finer meshes would appear all black at this resolution. $\rightarrow$, $\curvearrowright$ and $=$ denote solutions of a model on a growing domain, mapping of the solution on a FEM mesh, and solution mapped on a new mesh, respectively.

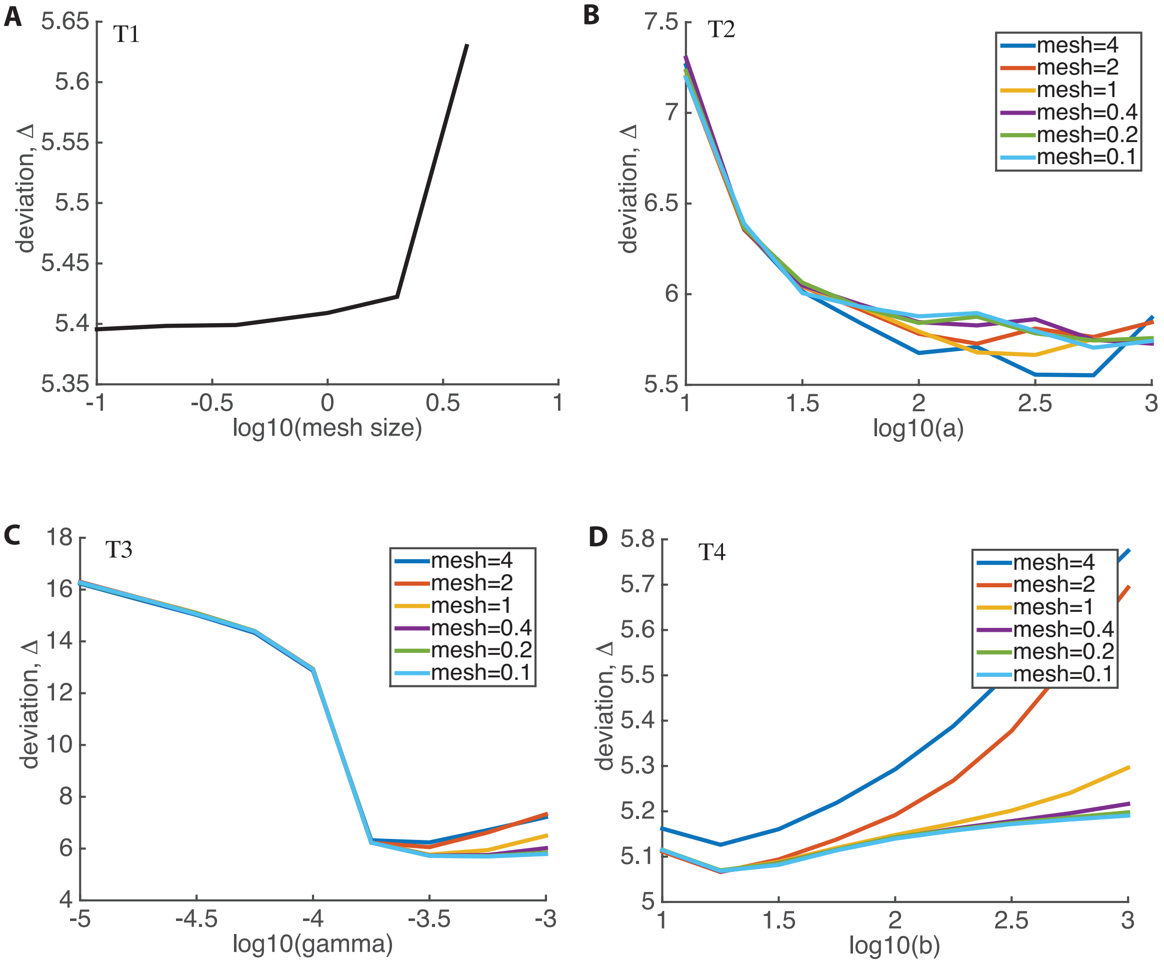

Supplementary Figure 19. Convergence Tests, wild type kidney. (A) Deviation, Δ versus mesh size calculated for the complete ligand-receptor based model, T1. (B) Deviation, Δ versus receptor production rate constant a, calculated for the model T2. (C) Deviation, Δ versus γ, calculated for the model T3. (D) Deviation, Δ versus b, calculated for the model T4. (B-D) Mesh sizes are indicated by the colorcode. All convergence plots were calculated for the time frame 20h.

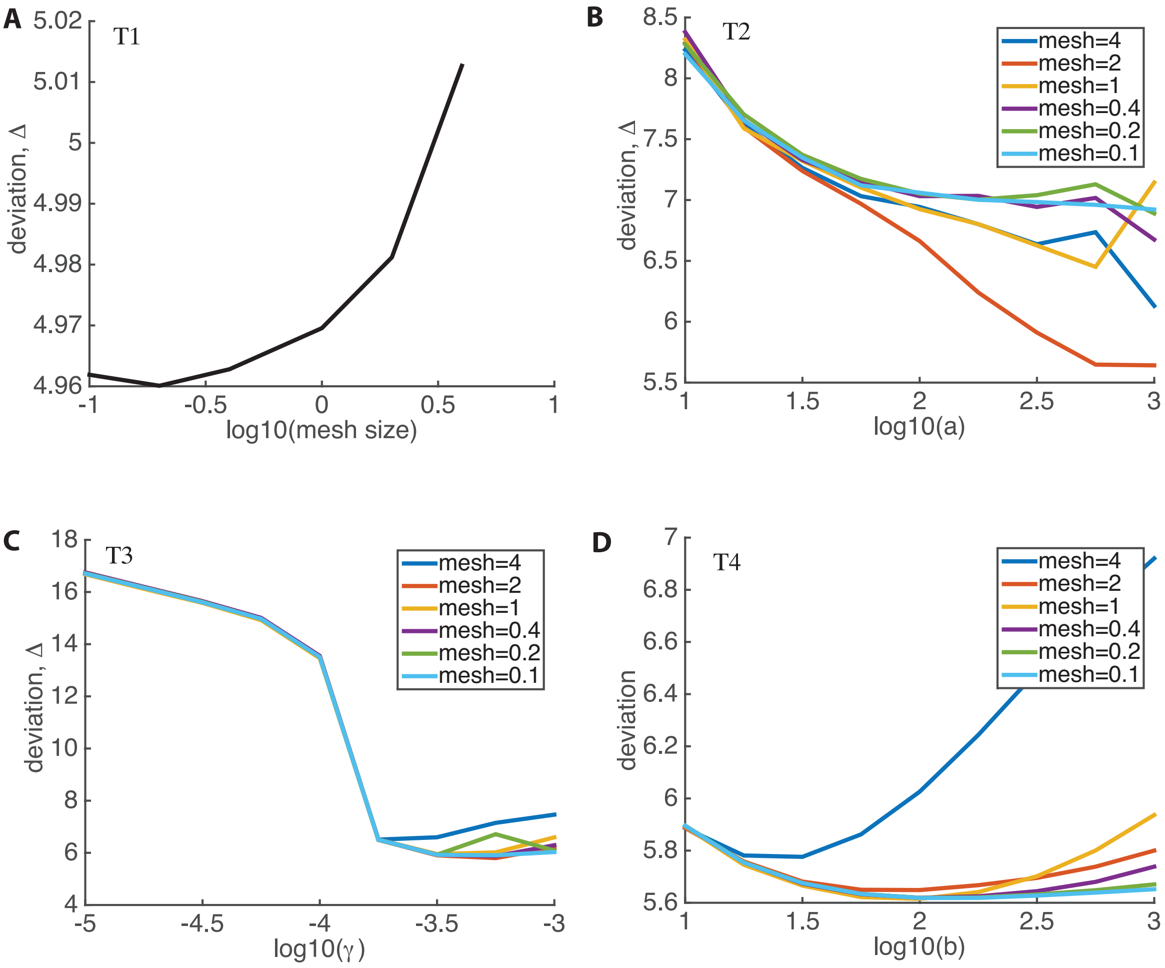

Supplementary Figure 20. Convergence Tests, FF kidney. (A) Deviation, Δ versus mesh size calculated for the complete ligand-receptor based model, T1. (B) Deviation, Δ versus receptor production rate constant a, calculated for the model T2. (C) Deviation, Δ versus γ, calculated for the model T3. (D) Deviation, Δ versus b, calculated for the model T4. (B-D) Mesh sizes are indicated by the colorcode. All convergence plots were calculated for the time frame 20h.

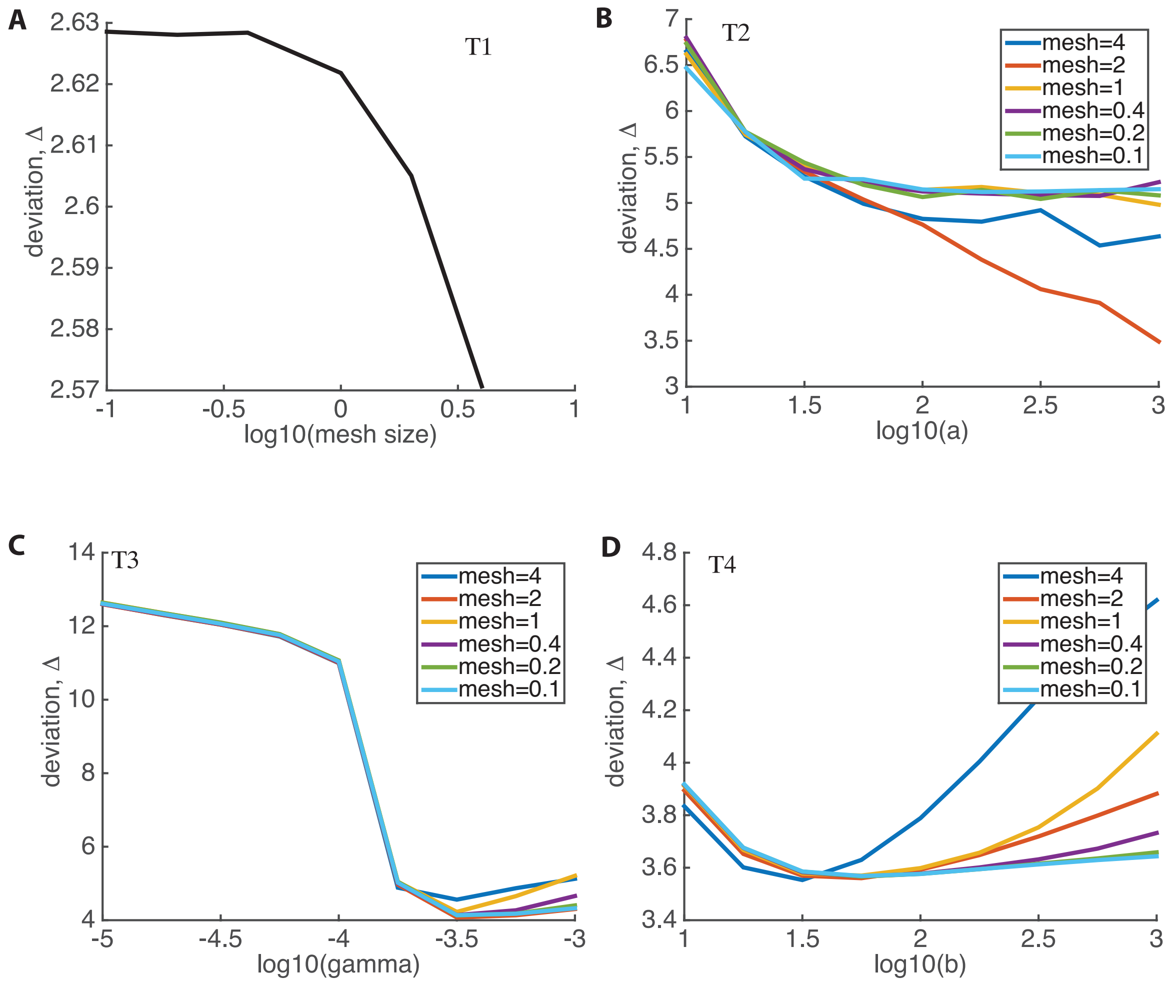

Supplementary Figure 21. Convergence Tests, FGS kidney. (A) Deviation, Δ versus mesh size calculated for the complete ligand-receptor based model, T1. (B) Deviation, Δ versus receptor production rate constant a, calculated for the model T2. (C) Deviation, Δ versus γ, calculated for the model T3. (D) Deviation, Δ versus b, calculated for the model T4. (B-D) Mesh sizes are indicated by the colorcode. All convergence plots were calculated for the time frame 20h.

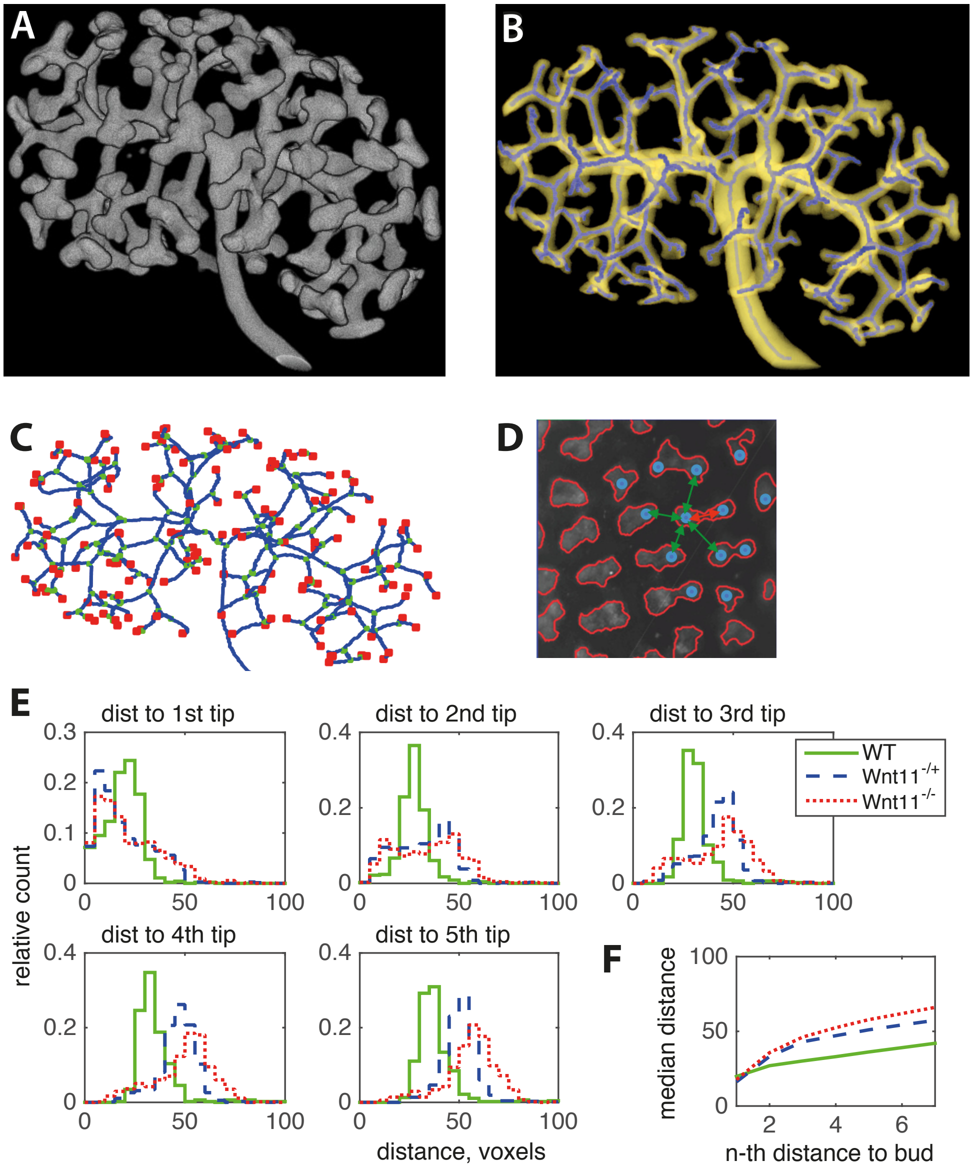

Supplementary Figure 22. Analysis of the Interbud Distances in wild type and Mutant Kidneys. (A) A 3D rendering of a segmented OPT image depicting a $Wnt^{+/-}$ kidney. (B) Skeleton (in blue) extracted from the segmented image depicted in (A). (C) Skeleton classified into end points (red), junctions (green), and slabs (blue). (D) Kidney tip and its neighbourhood. Red lines delineate the border of the ureteric epithelium. Blue circles depict kidney tips, the arrows indicate bud to bud distances. The red arrow indicates a distance between two tips that branch out from the same node. (E) Histograms of the distances of a bud tip to the next closest, 2nd closest, 3rd closest, 4th closest, and 5th closest bud tip in wild type and mutant kidneys. (F) Median distance to the n-th closest bud.

Supplementary References

1. Kurics, T., Menshykau, D. & Iber, D. Feedback, receptor clustering, and receptor restriction to single cells yield large turing spaces for ligand-receptor-based turing models. *Phys. Rev. E* **90**, 022716 (2014).

2. Murray, J. Mathematical biology. 3rd edition in 2 volumes: Mathematical biology: Ii. spatial models and biomedical applications. (2003).

3. Schindelin, J. *et al.* Fiji: an open-source platform for biological-image analysis. *Nat Methods* **9**, 676–682 (2012). 1081-8693.

4. Ollion, J., Cochennec, J., Loll, F., Escudé, C. & Boudier, T. Tango: a generic tool for high-throughput 3d image analysis for studying nuclear organization. *Bioinformatics* **29**, 1840–1841 (2013).

5. Otsu, N. A threshold selection method from gray-level histograms. *IEEE Transactions on Systems, Man, and Cybernetics* **9**, 62–66 (1979).

6. Landini, G. Auto threshold (2017). URL http://imagej.net/Auto_Threshold.

7. Arganda-Carreras, I. Skeletonize3d (2014). URL http://imagejdocu.tudor.lu/doku.php?id=plugin:morphology:skeletonize3d:start#skeletonize3d.

8. Arganda-Carreras, I., Fernández-González, R., Muñoz-Barrutia, A. & Ortiz-De-Solorzano, C. 3d reconstruction of histological sections: Application to mammary gland tissue. *Microscopy Research and Technique* **73**, 1019–1029 (2010).

9. Short, K. M. *et al.* Global quantification of tissue dynamics in the developing mouse kidney. *Dev Cell* **29**, 188–202 (2014).

10. Yuri. Ellipsoid fit (2015). URL https://mathworks.com/matlabcentral/fileexchange/24693-ellipsoid-fit.
