# Supplementary material for: Image-based modeling of kidney branching morphogenesis reveals GDNF-RET based Turing-type mechanism and pattern-modulating WNT11 feedback
Source: Nat Commun. 2019 Jan 16;10:239. doi: 10.1038/s41467-018-08212-8 (PMC6484223; doi:10.1038/s41467-018-08212-8)
Supplement: Supplementary file 3 — Description of Additional Supplementary Files [file 41467_2018_8212_MOESM3_ESM.docx]

**Description of Additional Supplementary Files**

File Name: Supplementary Movie 1

Description: A time-lapse movie of an in vitro culture of a wild type embryonic kidney undergoing branching morphogenesis. The scale bar corresponds to 200 µm.

File Name: Supplementary Movie 2

Description: A time-lapse movie of an in vitro culture of an Fgf10-/- (FF) mutant embryonic kidney undergoing branching morphogenesis. The scale bar corresponds to 200 µm.

File Name: Supplementary Movie 3

Description: A time-lapse movie of an in vitro culture of a Fgf10+/−; Gdnf+/−; Spry+/− (FGS) mutant embryonic kidney undergoing branching morphogenesis. The scale bar corresponds to 200 µm.

File Name: Supplementary Movie 4

Description: Extracted border and displacement field of the epithelium from a wild type embryonic kidney undergoing branching morphogenesis in an in vitro culture. The scale bar corresponds to 200 µm.

File Name: Supplementary Movie 5

Description: Extracted border and displacement field of the epithelium from an Fgf10-/- (FF) mutant embryonic kidney undergoing branching morphogenesis in an in vitro culture. The scale bar corresponds to 200 µm.

File Name: Supplementary Movie 6

Description: Extracted border and displacement field of the epithelium from a Fgf10+/−; Gdnf+/−; Spry+/− (FGS) mutant embryonic kidney undergoing branching morphogenesis in an in vitro culture. The scale bar corresponds to 200 µm.

File Name: Supplementary Movie 7

Description: The areas with high levels of receptor-ligand complexes (R2L) as predicted by the ligand-receptor based Turing mechanism recapitulate the areas of growth observed during wild type kidney branching morphogenesis. The scale bar corresponds to 200 µm.

File Name: Supplementary Movie 8

Description: In silico branching morphogenesis, wild type kidney.

File Name: Supplementary Movie 9

Description: The areas with high levels of receptor-ligand complexes (R2L) as predicted by the ligand-receptor based Turing mechanism recapitulate the areas of growth observed during Fgf10-/- (FF) mutant kidney branching morphogenesis. The scale bar corresponds to 200 µm.

File Name: Supplementary Movie 10

Description: The areas with high levels of receptor-ligand complexes (R2L) as predicted by the ligand-receptor based Turing mechanism recapitulate the areas of growth observed during Fgf10+/−; Gdnf+/−; Spry+/− (FGS) mutant kidney branching morphogenesis. The scale bar corresponds to 200 µm.

File Name: Supplementary Movie 11

Description: In silico branching morphogenesis, FF kidney.

File Name: Supplementary Movie 12

Description: In silico branching morphogenesis, FGS kidney.

File Name: Supplementary Movie 13

Description: Branching patterns simulated on a growing domain with the complete ligand-receptor model, T1.

File Name: Supplementary Movie 14

Description: Branching patterns simulated on a growing domain with the model with the additional positive feedback, T5.
