# Supplementary material for: Image-based modeling of kidney branching morphogenesis reveals GDNF-RET based Turing-type mechanism and pattern-modulating WNT11 feedback
Source: Nat Commun. 2019 Jan 16;10:239. doi: 10.1038/s41467-018-08212-8 (PMC6484223; doi:10.1038/s41467-018-08212-8)
Supplement: Supplementary file 18 — Reporting Summary [file 41467_2018_8212_MOESM18_ESM.pdf]

nature research

Corresponding author(s): *Double-blind peer review submissions: write DBPR and your manuscript number here instead of author names.*

Reporting Summary

Nature Research wishes to improve the reproducibility of the work that we publish. This form provides structure for consistency and transparency in reporting. For further information on Nature Research policies, see Authors & Referees and the Editorial Policy Checklist.

Statistical parameters

When statistical analyses are reported, confirm that the following items are present in the relevant location (e.g. figure legend, table legend, main text, or Methods section).

n/a	Confirmed	
☐	☒	The exact sample size (*n*) for each experimental group/condition, given as a discrete number and unit of measurement
☐	☒	An indication of whether measurements were taken from distinct samples or whether the same sample was measured repeatedly
☐	☒	The statistical test(s) used AND whether they are one- or two-sided<br>*Only common tests should be described solely by name; describe more complex techniques in the Methods section.*
☐	☒	A description of all covariates tested
☐	☒	A description of any assumptions or corrections, such as tests of normality and adjustment for multiple comparisons
☐	☒	A full description of the statistics including central tendency (e.g. means) or other basic estimates (e.g. regression coefficient) AND variation (e.g. standard deviation) or associated estimates of uncertainty (e.g. confidence intervals)
☐	☒	For null hypothesis testing, the test statistic (e.g. *F*, *t*, *r*) with confidence intervals, effect sizes, degrees of freedom and *P* value noted<br>*Give P values as exact values whenever suitable.*
☒	☐	For Bayesian analysis, information on the choice of priors and Markov chain Monte Carlo settings
☒	☐	For hierarchical and complex designs, identification of the appropriate level for tests and full reporting of outcomes
☒	☐	Estimates of effect sizes (e.g. Cohen's *d*, Pearson's *r*), indicating how they were calculated
☐	☒	Clearly defined error bars<br>*State explicitly what error bars represent (e.g. SD, SE, CI)*

Our web collection on statistics for biologists may be useful.

Software and code

Policy information about availability of computer code

Data collection	N/A
Data analysis	NRecon (Skyscan), FiJi, COMSOL Multiphysics 4.x, MATLAB 8.4

For manuscripts utilizing custom algorithms or software that are central to the research but not yet described in published literature, software must be made available to editors/reviewers upon request. We strongly encourage code deposition in a community repository (e.g. GitHub). See the Nature Research guidelines for submitting code & software for further information.

Data

Policy information about availability of data

All manuscripts must include a data availability statement. This statement should provide the following information, where applicable:

- Accession codes, unique identifiers, or web links for publicly available datasets
- A list of figures that have associated raw data
- A description of any restrictions on data availability

The data that support the findings of this study are available from the corresponding author upon reasonable request.

Field-specific reporting

Please select the best fit for your research. If you are not sure, read the appropriate sections before making your selection.

☒ Life sciences ☐ Behavioural & social sciences ☐ Ecological, evolutionary & environmental sciences

For a reference copy of the document with all sections, see nature.com/authors/policies/ReportingSummary-flat.pdf

Life sciences study design

All studies must disclose on these points even when the disclosure is negative.

Sample size	The sample size was not determined up front, as information about the effect size was not available. The sample size for the determination of the inter-bud distance was driven by the number of samples necessary to cover the tested range of kidney volumes.
Data exclusions	Data (3D Images) were excluded from the analysis only if the image analysis algorithm failed to provide a reasonable segmentation.
Replication	Organ culture experiments were performed as independent replicates of three. For the bead experiments, at least 5 control and 10 GDNF samples were cultured per experiment.<br>Wnt11 mutant and wild type kidney sample sizes are indicated in table 1 of the manuscript.
Randomization	For GDNF (bead) experiments, kidneys were dissected from embryos of a single litter and randomly assigned to control or +GDNF group.
Blinding	No blinding was done as kidney phenotypes are obvious and control and mutant/treated samples can be easily distinguished from controls.

Reporting for specific materials, systems and methods

Materials & experimental systems

n/a	Involved in the study
☒	☐ Unique biological materials
☐	☒ Antibodies
☒	☐ Eukaryotic cell lines
☒	☐ Palaeontology
☐	☒ Animals and other organisms
☒	☐ Human research participants

Methods

n/a	Involved in the study
☒	☐ ChIP-seq
☒	☐ Flow cytometry
☒	☐ MRI-based neuroimaging

Antibodies

Antibodies used	Primary antibodies:<br>Phospho-p44/42 MAPK (Erk1/2) (Thr202/Tyr204) XP® Rabbit mAb, Cell Signaling, Cat#4370, clone name: D13.14.4E, Lot#15<br>Monoclonal Anti-Cytokeratin, pan antibody produced in mouse, Sigma-Aldrich, Cat#C1801, clone name: PCK-26<br>Monoclonal Anti-E-cadherin Antibody produced in rat, Sigma-Aldrich, Cat#MABT26, clone name: DECMA-1, Lot#046M4853V<br><br>Secondary antibodies:<br>Donkey anti-Rabbit IgG (H+L) Highly Cross-Adsorbed Secondary Antibody, Alexa Fluor 555, Thermo-Fisher Scientific, Cat#A31572, LOT#1671993<br>Donkey anti-Rat IgG (H+L) Highly Cross-Adsorbed Secondary Antibody, Alexa Fluor 488, Thermo-Fisher Scientific, Cat#A21208, Lot#1744717<br>Goat anti-Mouse IgG1 Cross-Adsorbed Secondary Antibody, Alexa Fluor 555, Thermo-Fisher Scientific, Cat#A21127, Lot#1640321
Validation	The manufacturer's validation for phosphoErk1/2 can be found here: https://media.cellsignal.com/pdf/4370.pdf The Ab is suitable for Immunofluorescence and reactive to Mouse. For this study, staining was performed simultaneously in murine kidneys and lungs and yielded comparable results for pERK-stained domains at bud tips for both organs.<br>Anti-Cytokeratin, pan cross reacts with mouse cytokeratins and is recommended for use in immunofluorescence assays (https://www.sigmaaldrich.com/content/dam/sigma-aldrich/docs/Sigma/Datasheet/c1801dat.pdf)<br>Anti-E-cadherin cross reacts with mouse and is recommended for use in immunofluorescence assays (http://www.merckmillipore.com/CH/de/product/Anti-E-cadherin-Antibody-clone-DECMA-1,MM_NF-MABT26?bd=1)<br><br>Secondary antiboy validation / information can be found on the manufacturer's website: https://www.thermofisher.com/antibody/product/Donkey-anti-Rabbit-IgG-H-L-Highly-Cross-Adsorbed-Secondary-Antibody-Polyclonal/A-31572

https://www.thermofisher.com/antibody/product/Donkey-anti-Rat-IgG-H-L-Highly-Cross-Adsorbed-Secondary-Antibody-Polyclonal/A-21208 https://www.thermofisher.com/antibody/product/Goat-anti-Mouse-IgG1-Cross-Adsorbed-Secondary-Antibody-Polyclonal/A-21127

Animals and other organisms

Policy information about studies involving animals; ARRIVE guidelines recommended for reporting animal research

Laboratory animals	Mus musculus; 129S.Cg-Tg(Hoxb7-Venus)17Cos/J, 129S.Cg-Tg(Hoxb7-EGFP)33Cos/J, Wnt11(tm1a(KOMP)wtsi) was maintained in C57/Bl6J background; Fgf10, GDNF and Sprouty1 lines were maintained in mixed a background (129S1/SvmJ:C57BL/6). Sex: Embryos, Age: E11.5-14.5
Wild animals	No wild animals were used.
Field-collected samples	No field-collected samples were used.
